# Endurance-oriented training program with children and adolescents on maintenance hemodialysis to enhance dialysis efficacy—DiaSport

**DOI:** 10.1007/s00467-021-05114-8

**Published:** 2021-06-12

**Authors:** Markus Feldkötter, Sarah Thys, Anne Adams, Ingrid Becker, Rainer Büscher, Martin Pohl, Raphael Schild, Lars Pape, Claus Peter Schmitt, Christina Taylan, Simone Wygoda, Günter Klaus, Henry Fehrenbach, Carmen Montoya, Martin Konrad, Heiko Billing, Bettina Schaar, Bernd Hoppe

**Affiliations:** 1 Pediatric Nephrology Unit, University Children’s Hospital Zurich, Zurich, Switzerland; 2 Institute of Sport Science, Bundeswehr University Munich, Munich, Germany; 3 Institute of Medical Statistics and Computational Biology, University Hospital of Cologne, Cologne, Germany; 4 Pediatric Nephrology, Pediatrics II, University of Duisburg-Essen, Essen, Germany; 5 Department of General Pediatrics, Adolescent Medicine and Neonatology, Medical Center – University of Freiburg, Faculty of Medicine, University of Freiburg, Freiburg, Germany; 6 Pediatric Nephrology, University Children’s Hospital, University Medical Center Hamburg-Eppendorf, Hamburg, Germany; 7 Department of Pediatric Kidney, Liver and Metabolic Diseases, Hannover Medical School, Hannover, Germany; 8 Division of Pediatric Nephrology, Center for Pediatric and Adolescent Medicine, Heidelberg, Germany; 9 Pediatric Nephrology, Children’s and Adolescents’ Hospital, University of Cologne, Cologne, Germany; 10 KfH Center of Pediatric Nephrology, St Georg Hospital, Leipzig, Germany; 11 KfH Center of Pediatric Nephrology, Department of Pediatric Nephrology, Marburg, Germany; 12 Pediatric Nephrology, Children’s Hospital, Memmingen, Germany; 13 KfH Center of Pediatric Nephrology, Children’s Hospital Munich Schwabing, Munich, Germany; 14 Department of General Pediatrics, University Children’s Hospital, Münster, Germany; 15 University Children’s Hospital, Tübingen, Germany; 16 Children’s Hospital, University of Bonn, Bonn, Germany; 17 Kindernierenzentrum Bonn, Bonn, Germany

**Keywords:** Sport, Hemodialysis, Endurance training, Children, Adolescents, spKt/V

## Abstract

**Objective:**

Pediatric patients spend significant time on maintenance hemodialysis (HD) and traveling. They are often not capable of participating in sports activities. To assess the effects of exercise training during HD on dialysis efficacy in children and adolescents, we set up a multi-center randomized controlled trial (RCT).

**Methods:**

Patients on HD, age 6 to 18 years, were randomized either to 3× weekly bicycle ergometer training or to no training during HD for 12 weeks. Change in single-pool Kt/V (spKt/V) was the primary outcome parameter.

**Results:**

We randomized 54 patients of whom 45 qualified (23 in the intervention and 22 in the waiting control group, 14.5 ± 3.01 years, 32 male and 13 female) for the intention-to-treat (ITT) population. Only 26 patients finished study per-protocol (PP). Training was performed for an average of 11.96 weeks (0.14–13.14) at 2.08 ± 0.76 times per week and for a weekly mean of 55.52 ± 27.26 min. Single-pool Kt/V was similar in the intervention compared to the control group (1.70 [0.33] vs. 1.79 [0.55]) at V0 and (1.70 [0.36] vs. 1.71 [0.51]) at V1; secondary endpoints also showed no difference in both ITT and PP analysis. No significant adverse events were reported. No bleeding or needle dislocation occurred in 1670 training sessions.

**Conclusions:**

Intradialytic bicycle training is safe, but does not improve dialysis efficacy and physical fitness. However, the study can be considered underpowered, particularly because of high dropout rates. Future studies need better strategies to increase motivation and compliance and other more effective/intensive exercise measures should be evaluated.

**Trial registration:**

The trial was registered in ClinicalTrials.Gov (Clinicaltrials.gov identifier: NCT01561118) on March 22, 2012.

**Supplementary Information:**

The online version contains supplementary material available at 10.1007/s00467-021-05114-8.

## Introduction

Patients with stage 5 chronic kidney disease (CKD 5) undergoing hemodialysis (HD) have lower cardiorespiratory fitness, poorer health-related quality of life (HRQoL), and a functional impairment compared with age-matched controls [1]. Their physical endurance capacity is lower than that of healthy peers. Due to HD-associated time constraints and exhaustion after HD, patients are frequently not able to participate in regular sports activities [2, 3]. In addition, HD treatment is extensively time-consuming and is physically exhausting [3, 4]. However, intradialytic exercise is feasible and may have a positive effect in patients on HD [5, 6]. A center-based HD treatment provides opportunities to combine monitored and guided exercise with medical treatment, i.e., to effectively use the time spent on HD [5, 7]. Potential complications can be recognized and treated promptly [8]. Even though (intradialytic) exercise training is recommended in patient care guidelines, it is still not a routine treatment for patients undergoing HD. Reasons for that may be no best training modality defined, a lack of interest in training, and/or insufficient manpower to support training during HD [9, 10].

A systematic literature review on exercise during HD showed improvements of cardiorespiratory fitness, physical performance, and self-reported physical function in HRQoL questionnaires [11]. But studies addressing the issue of fitness and exercise performance were usually performed in adult CKD 5 patients [2]. Direct translation of data from adults to children and adolescents is not applicable, because many adults have complicating co-morbidities, including cardiovascular disease and diabetes mellitus. Nevertheless, the causes of exercise limitations may be similar in children and adults. In pediatric and adolescent patients, lack of time due to time spent in school, time on HD, and traveling to/from HD are additional limiting factors when considering exercise studies in a pediatric population.

Several studies provided evidence for an increase of dialysis efficacy (15–25% increase in urea clearance) with a single bout of exercise during dialysis [12, 13]. A significant difference of single-pool Kt/V calculated by a second-generation logarithmic equation was shown in one study in adult HD patients [14]. Here, it was observed that the increased muscle blood flow and greater amount of open capillary surface area in working muscles resulted in a greater flux of urea and associated toxins from the tissue to the vascular compartment for subsequent removal at the dialyzer [14]. Other studies in adults failed to demonstrate a significant increase in dialysis efficacy, most likely due to small patient numbers, or the used exercise intensity [15, 16]. Respective pediatric data have not yet been obtained.

We therefore designed a study to determine the effects of long-term bicycle ergometer training on dialysis efficacy and physical performance in children and adolescents on maintenance hemodialysis.

### Methods

**Fig. 1:**
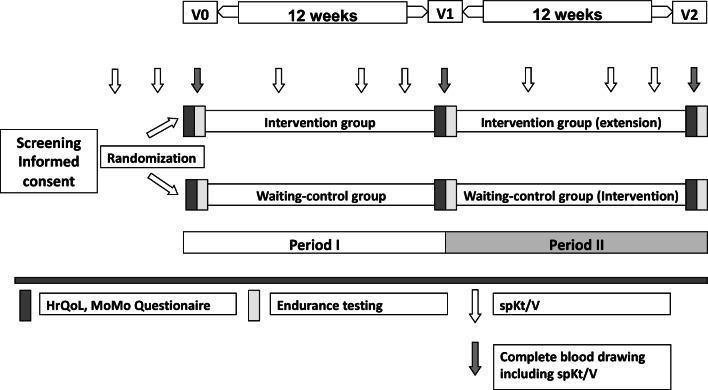
Study schedule: After screening and randomization of the patient, three times weekly bicycle ergometer training during hemodialysis for up to 50 to 70 min (warm up, endurance training, cool down) duration and with 70–80% of the patient-specific maximum workload during 12 weeks was performed twice in the intervention group (periods I and II). In the waiting control group, no ergometer training was performed during the first 12 weeks (period I); training was then performed in intervention period II

In this prospective, randomized, multi-center clinical trial, patients were randomized into either the intervention or the waiting control group (Fig. 1) using a computer-based randomization list generated by the trial statistician. The randomization was performed with a 1:1 ratio stratified by center. Concealed envelopes with subsequent numbers were kept in a locked drawer on the switchboard and were withdrawn consecutively after the baseline tests. A blinding was not feasible due to the study intervention.

Primary inclusion criteria were as follows: (1) medically stable patients on HD treatment for ≥ 3 months and aged between ≥ 6 and ≤ 19 years at the time of randomization, and (2) stable dialysis conditions, as determined by serum parameters and single-pool Kt/V (> 1.2), for at least 4 weeks prior to intervention and an informed consent. Exclusion criteria were participation in another interventional study; uncontrolled hypertension or hypotension; recurrent, uncontrolled epileptic seizures; any heart disease; other severe primary or secondary diseases known as a contraindication for endurance training; and already planned medical intervention, for example, living donor kidney transplantation or any other surgery within the next 3 months after randomization, which would lead to a discontinuation of training for more than 2 weeks. During the study period, the participating centers were asked to maintain the same dialysis modality including unchanged blood flow, dialysis time, and dialyzer.

Single-pool Kt/V calculation was performed with the formula of Daugirdas [17]. We had experienced that single-pool Kt/V calculated by this formula has a very small variability in our maintenance HD patients. From our unpublished data, the mean improvement of single-pool Kt/V for a group undergoing ergometer training was about 0.20 with an SD of 0.25, yielding a standardized effect of 0.8. Using the error probabilities of *α* = 0.05 and *β* = 0.8, the sample size needed to show such an effect would be *n* = 26 in each group, i.e., 52 in total. A dropout rate of 20% was expected. Therefore, we planned to randomize 66 patients. We expected a screen failure rate of up to 50%. This would have made screening of 132 patients necessary to assess eligibility (taking mal-compliance, disease-related problems, transplantation, or withdrawal from study into consideration).

Study intervention was a three times weekly bicycle ergometer training for 12 weeks (Fig. 1). It started during the first 30 min of HD, with a duration of up to 50–70 min (warm up, endurance training, and cool down) and with 70–80% of the patient-specific maximum workload. The exercise intervention was performed using a bicycle ergometer (Medical8i ergo_bike, Daum Electronic, Fuerth, Germany) positioned beside the patient’s dialysis chair or bed. Patients sat on the ergobike during dialysis and performed their training. We opted for a one-time intervention, as it was accompanied by one trainer at a time. Splitting it into two trainings would have made this already complex approach even more difficult, and it would certainly have been more challenging to motivate the participants.

For all training sessions, an individual training scheme was designed based on the endurance testing scheduled at the study visits. During the training sessions, the participants were supervised and monitored by a well-trained assistant student and the trial sports scientist monitored the intervention.

For the intervention group, exercise training was performed for 12 weeks (36 sessions) in trial period I, followed by a 12-week prolongation (36 training sessions) in trial period II. The waiting control group underwent standard HD treatment without any supervised exercise for 12 weeks in trial period I, followed by 12 weeks of training (36 training sessions) according to the intervention group in trial period II.

Ergometric testing was always performed on dialysis days prior to start of HD, to minimize travel times. Cardiopulmonary exercise testing was performed at baseline and after 3 and 6 months, respectively. The test was performed by ergospirometry with workload levels increasing every minute, according to Godfrey [18]. The subjects started with a watt setting corresponding to half their weight in kilograms. The workload was increased by 10 watts/min until subjective exhaustion. The following parameters were measured: time in minutes, number of levels, work in watts, and heart rate (initial heart rate, heart rate during the test, peak heart rate, and recovery heart rate after 4 min) as well as respiratory parameters (VO2-peak, VO2-peak/kg, respiratory exchange ratio (RER)). Lactate values and perceived exertion according to BORG completed the measured parameters [19, 20].

Albumin, alkaline phosphatase, calcium, cholesterol, C-reactive protein, ferrum, protein, ferritin, urea acid, HDL-cholesterol, sodium, transferrin, triglyceride, PTH, creatinine, phosphate, and urea were measured by standard methods in a central routine clinical laboratory (Labor Enders und Partner, Stuttgart). Blood count including hemoglobin and potassium was measured at the local laboratory as these parameters are not stable and inappropriate to ship.

Quality of life was determined by the PedsQL questionnaire for patients and their parents. The physical fitness and physical activity were measured by the questionnaire “Motorik-Modul” (MoMo): Whenever needed, the assistant student and/or the trial sports scientist helped in filling out the questionnaires.

The primary endpoint was the change of single-pool Kt/V measured at week 12 (V1) compared to baseline (V0). Single-pool Kt/V is the standard measure to assess dialysis efficacy. As adequate dialysis efficacy is the primary aim of every dialysis treatment and the single-pool Kt/V is the best way to measure efficacy, it has an important clinical relevance for the patient.

To evaluate efficiency and test accuracy, comparison of the changes in single-pool Kt/V from week 0 to week 12 between the treatment groups was performed. The primary endpoint was evaluated by an analysis of covariance (ANCOVA) with the fixed factors treatment group and center and single-pool Kt/V at week 0 baseline as covariates. Missing values were replaced by the LOCF (last observation carried forward) imputation method. For the waiting control group (group II), the changes in periods 1 (no intervention) and 2 (training intervention) were compared by a paired *t* test. In the intervention group (group I), the second intervention period was compared to the first by descriptive statistics to assess the effects of prolonged maintenance of training.

The secondary endpoints included change of the possible workload (maximum physical performance), quality of life, change of solute removal during HD, change of solute removal in the two compartment model, change in inflammation, nutritional status, and bone metabolism (body composition monitoring (BMC)).

The primary efficacy analysis was based on a modified intention-to-treat (mITT) population, including all randomized patients for whom one single-pool Kt/V at baseline and at least one value after baseline were documented. Patients who dropped out because of kidney transplantation or other reasons were excluded from this mITT set. Additionally, a per-protocol (PP) analysis was performed.

**Fig. 2:**
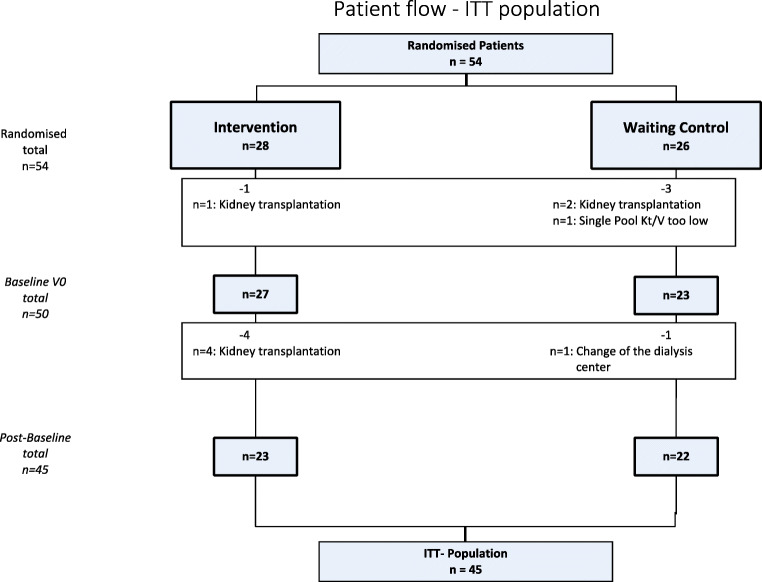
Patient flow intention-to-treat (ITT) population

**Fig. 3:**
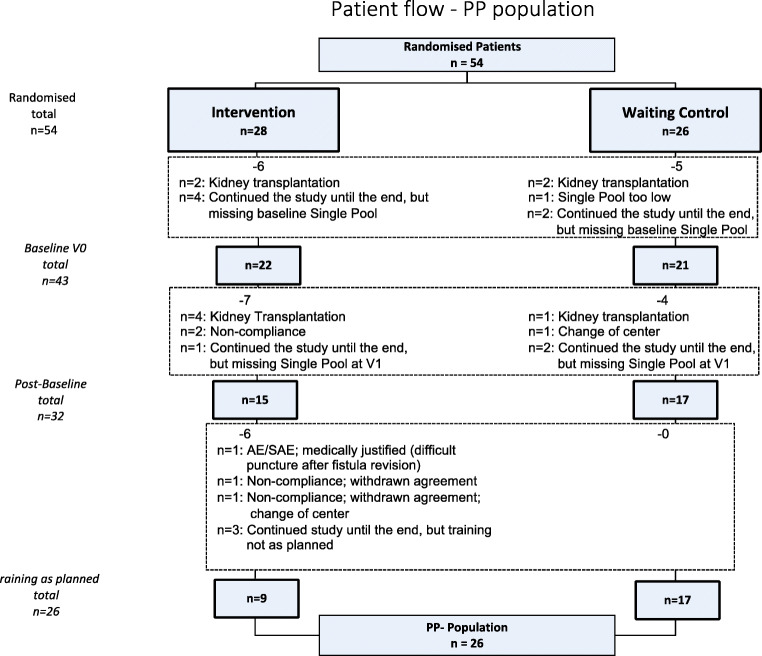
Patient flow per-protocol (PP) population

Criteria for the PP population analysis were as follows: all three single-pool Kt/V prior and to V0 and V1 and at least 80% of training performed during period 1 with a maximal interruption of 7 days in the intervention group (see Figs. 2 and 3).

For safety evaluations, cross tables and listings of adverse events were used.

The secondary endpoints were analyzed by appropriate ANCOVA models, non-parametric or chi-square tests, and descriptive statistics. Data analysis was performed using SAS 9.4 (SAS Institute Inc., Cary, NC, USA).

For monitoring, one pre-study selection visit per site for all of the study sites, one initiation visit per site, and one closing monitoring visit for all active study sites were planned.

The original protocol of the DIASPORT trial was approved by the Ethics Committees of the University of Cologne (11-282) and by all participating centers, and written informed consent was obtained from each participant and/or their legal guardian before study. The trial was registered in ClinicalTrials.Gov (Clinicaltrials.gov identifier: NCT01561118).

## Results

**Fig. 4:**
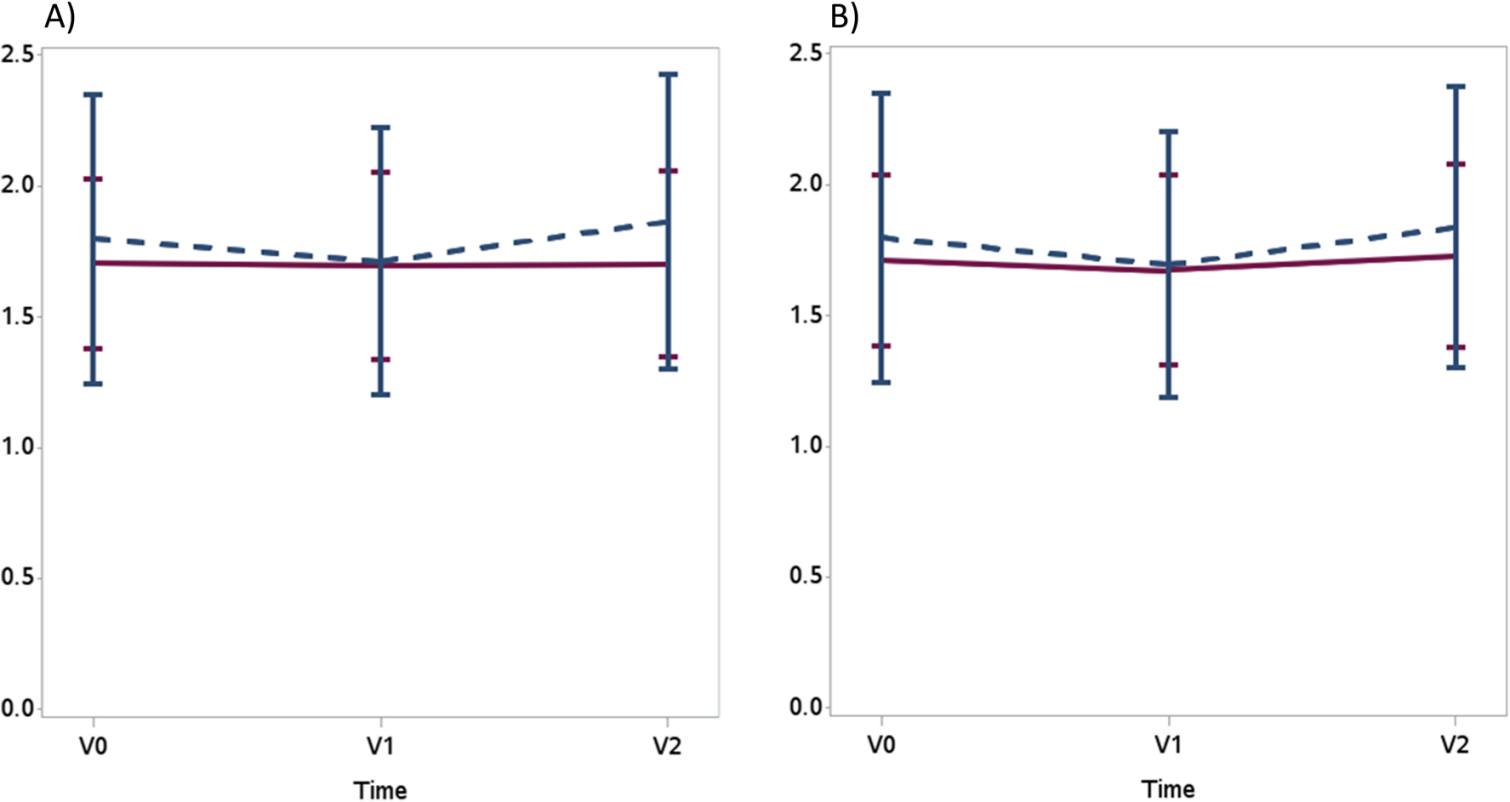
Primary endpoint: Mean single-pool Kt/V at different testing points, ITT (intention-to-treat population), LOCF (last observation carried forward). V0 baseline, V1 post first intervention period (12 weeks), V2 post second intervention period (24 weeks)

There was no significant difference of the primary endpoint single-pool Kt/Vin the intervention group compared to the waiting control group. The single-pool Kt/V at baseline (V0) was 1.70 (0.33) in the intervention vs. 1.79 (0.55) in the waiting control group, with a median of 1.64 (range 1.18–2.37) vs. 1.79 (1.02–3.12), respectively. No differences were found at V1 and V2 (Table 1; Fig. 4; supplemental material). Dialysis time in both groups at V0 was 257 min (23.03) vs. 259.95 min (22.46).

**Table 1:** Single-pool Kt/V data V0–V2 (ITT population)

	Intervention group	Control group	*p* value
V0	1.70 (0.33)	1.80 (0.55)	0.4922
V1	1.70 (0.36)	1.71 (0.51)	0.9081
V2	1.70 (0.36)	1.86 (0.56)	0.3605

**Fig. 5:**
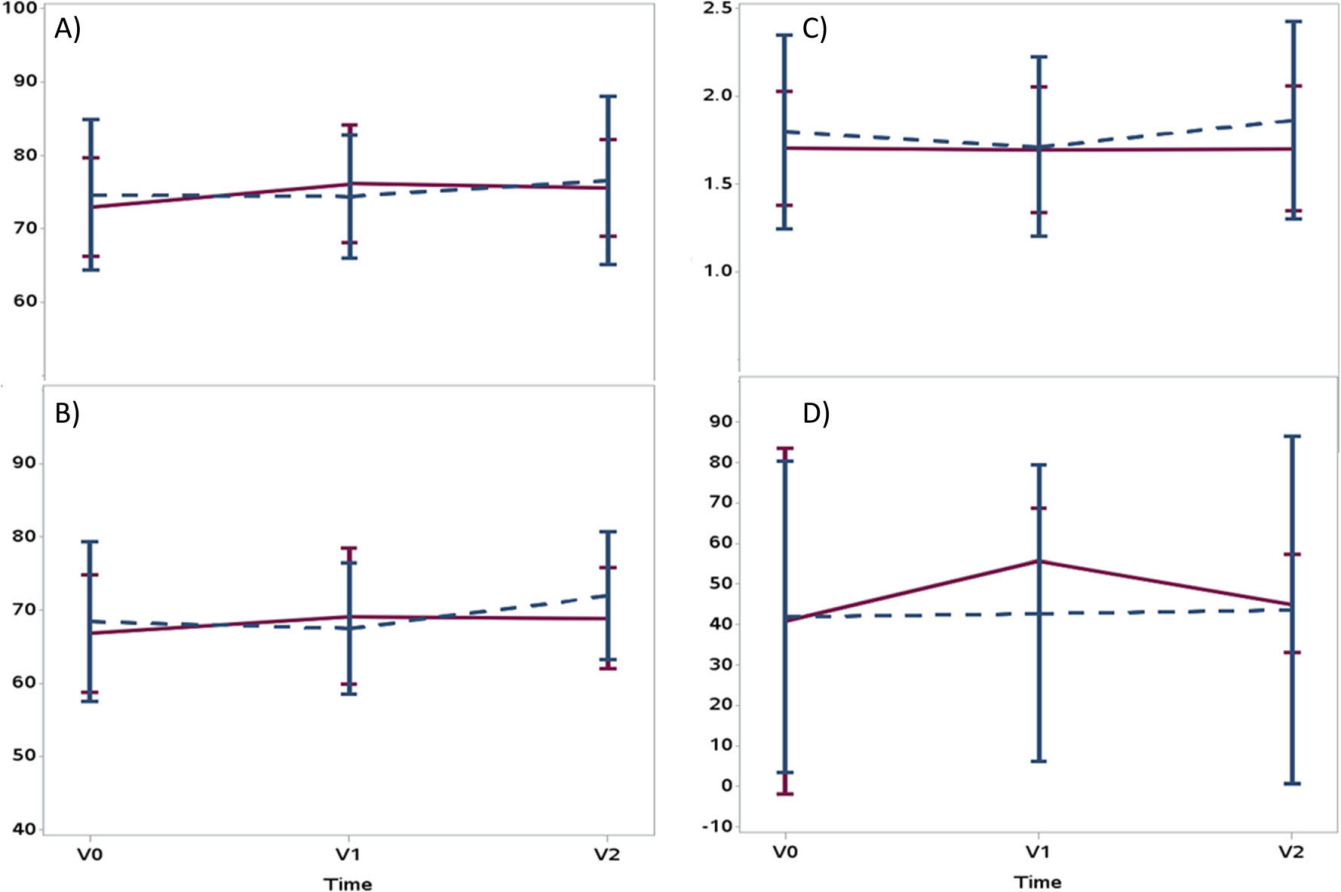
Exemplary secondary endpoint: mean elimination of low molecular substances—no changes between V0 and V2 for blood urea, serum creatinine, serum phosphate, and mean double-pool Kt/V

With regard to all secondary endpoints, no significant differences were found (see Table 2, Fig. 5, and supplemental material: statistical report and respective figures).

**Table 2:** Primary endpoint and secondary endpoints (V1 vs. V0) in the intention-to-treat (ITT) population (value and in brackets standard deviation)

	Training group (TG)		Control group (CG)		*p* value
	V0	V1	V0	V1	
Primary endpoint					
Single-pool Kt/V	1.70 (0.33)	1.70 (0.36)	1.80 (0.55)	1.71 (0.51)	0.2245
Secondary endpoints					
Possible workload (max. physical performance)					
Maximum power (watt)	78.6 (27.4)	83.8 (32.5)	77.8 (21.5)	79.2 (23.6)	0.1699
Pulse (1/min)	83.7 (19.4)	83.6 (19.1)	81.1 (16.4)	87.3 (16.3)	0.0799
Quality of life (PedsQL)					
Total score parents	69.7 (21.2)	74.0 (11.7)	75.0 (15.4)	81.2 (7.1)	0.3204
Total score child	74.9 (15.7)	77.8 (12.3)	81.3 (11.8)	80.1 (10.9)	0.2853
Change of solute removal during HD					
Reduction of urea (%)	73.0 (6.8)	76.2 (8.0)	74.6 (10.3)	74.4 (8.4)	0.5133
Reduction of creatinine (%)	66.8 (8.0)	69.1 (9.3)	68.4 (10.9)	67.5 (9.0)	0.5110
Reduction of phosphate (%)	40.8 (42.7)	55.7 (13.1)	41.9 (38.5)	42.7 (36.6)	0.0869
Reduction of potassium (%)	27.6 (9.7)	25.8 (7.6)	26.8 (12.5)	24.6 (11.3)	0.9054
Change of solute removal in a two compartment model (TG *N* = 15; CG *N* = 18)					
Double-pool Kt/V	1.5 (0.22)	1.5 (0.26)	1.4 (0.39)	1.5 (0.39)	0.4985
Change in inflammation					
C-reactive protein (mg/L)	4.0 (10.9)	2.3 (3.2)	4.2 (5.9)	3.7 (4.6)	0.8253
Nutritional status and bone metabolism					
Albumin (g/L)	43.2 (3.3)	41.4 (5.5)	43.0 (3.3)	43.2 (3.3)	0.3115
Alkaline phosphatase (U/L)	179.2 (92.0)	179.9 (99.4)	204.4 (111.7)	231.5 (179.5)	0.9104
Parathyroid hormone (PTH) (ng/L)	282.3 (224.4)	318.4 (206.8)	229.9 (167.1)	250.8 (220.7)	0.6012
Body composition monitoring					
Total body water (L)	28.0 (9.9)	27.1 (9.1)	26.1 (5.9)	26.2 (7.0)	0.8396
Fat-free mass (kg)	17.8 (2.6)	17.7 (3.8)	17.2 (0.9)	21.1 (6.9)	0.4459
Fat mass (kg)	9.7 (7.4)	9.4 (7.1)	9.5 (6.4)	10.0 (5.7)	0.8406
Overhydration (L)	0.8 (1.6)	0.9 (1.1)	1.0 (1.0)	0.9 (1.4)	0.8467

Based on the sample size calculation, 66 patients should have been included in the study, but only 54 patients were randomized (for patient characteristics, see Table 3). The reduced inclusion was due to competing studies and lack of patients’ interest, but also worries about the safety of training on a bike may have played a role (despite maximal support during the study intervention).

**Table 3:** Baseline patient characteristics in DiaSport according to intervention and waiting control group

Characteristics	Intervention group	Waiting control group	*p* value
	23	22	
Gender			
Male	19	13	0.1075
Female	4	9	
Prior kidney transplantation			
No	18	16	0.7381
Yes	5	6	
Dialysis therapy			
< 1 year	18	16	0.1159
> 1 year	5	6	
Dialysis access			
Catheter	4	6	0.6937
AV fistula	17	15	
Both	2	1	
Age			
< 12 years	6	2	0.2427
> 12 years	17	20	
Mean age, years (SD)	14.0 (3.44)	15.1 (2.45)	0.2286
Height, cm (SD)	153.8 (21.34)	155.6 (15.68)	0.7411
Weight, kg (SD)	48.1 (18.16)	45.6 (12.50)	0.5940
BMI, kg/m^2^ (SD)	19.6 (4.03)	18.5 (2.41)	0.2576
Single-pool Kt/V (SD)	1.70 (0.33)	1.79 (0.55)	0.4922
Reduction of phosphate, (%) (SD)	40.8 (42.73)	41.9 (38.50)	0.9273
Hemoglobin, g/dL (SD)	11.2 (1.71)	11.2 (1.33)	0.9138

The evaluable ITT population consisted of 45/54 patients, 23 in the intervention group and 22 in the waiting control group, respectively. The PP population consisted of only 26 patients, 9 in the intervention group and 17 in the waiting control group (see Fig. 2).

Reasons for exclusion of patients from the study in both the ITT and PP populations were as follows: kidney transplantation (7), change of dialysis center (1), and in one case a protocol violation as the single-pool KT/V > 1.2 at inclusion was not fulfilled (1). Additional patients were excluded from the PP because of kidney transplantation (2), missing single-pool KT/V values (9), non-compliance (2), agreement withdrawn (2), training not as planned (3), and 1 adverse event (AE) (revision of arteriovenous fistula).

There was no significant difference in number and severity of AEs and serious adverse events (SAEs) during the training period in both groups and the waiting control period. In particular, no bleeding or dislocation of the dialysis needles occurred during the 1670 training sessions.

One initiation visit per site was performed for 14 study sites. All centers were finally monitored and got a closing monitoring visit.

## Discussion

This study was unable to show a significant effect on the primary endpoint, amelioration of single-pool Kt/V, the most important biochemical criterion for evaluating dialysis quality. Secondary endpoints may be of more value per individual patient, but, unfortunately, no significant improvements were seen here either. This was definitively unexpected and of course needs explanation. The patients were dialyzed according to the standard operating procedures of the German Working Group on Pediatric Dialysis [21]. We therefore have to consider that the routine care taking of pediatric patients on maintenance dialysis, here especially HD, was already at a high standard level before the study was started. This is expressed by high single-pool Kt/V levels at V0 for both study groups, and also at V1 for the waiting control group. Hence, the margin of possible improvement per se was so tiny that already the extremely good single-pool Kt/V before the start of the study impeded a positive study outcome. Nevertheless, as even no secondary endpoint aim was reached, we still have to consider other fundamental reasons.

Patients on maintenance HD spend significant time for treatment and travel to and from dialysis facilities, which may add up to a total of about 1000 h per year. This is especially the case in pediatric and adolescent patients, as there are only few facilities per country and travel times can be long. Hence, they lack spare time to participate in social and/or sports events. Also, chronic HD patients are frequently too exhausted to train after HD, have a lower endurance, and are not necessarily motivated to perform sports to improve their endurance capacity [1, 2]. Therefore, reductions of functional and cardiopulmonary capacity are obvious and lead to reduced activities in daily life and also to an increase of the mortality risk [3–6].

Studies focused on activity and exercise in adult maintenance HD patients have demonstrated a positive impact of sports on patients’ physical performance [1, 8, 22]. Similarly, exercise intervention during dialysis in adults alleviated causes and symptoms of reduced exercise capacity [7, 9, 23]. These studies have demonstrated that training during dialysis is regarded as safe, effective, and practical [1, 2] and stimulated interest to perform comparable studies in children and adolescents with CKD [10]. The possibility of an exercise program outside of dialysis was evaluated by a Dutch study focusing on the impact of a community-based exercise program in children with CKD 5. It was shown that a training program led to an increase of muscle mass and exercise capacity in those who finished the program, but the dropout rate was high and the program was not feasible for most HD children [24].

Nevertheless, it was observed that children and adolescents benefit from a training intervention leading to an increased aerobic capacity, such as muscle growth [2]. Endurance training during dialysis was indeed possible and safe in pediatric and adolescent patients, but a specific and individualized training control was extremely important [10]. However, it still has to be evaluated whether an appropriate training for children and adolescents with a high demand character is doable. It became clear that training during dialysis in children and adolescents should address specific individual challenges with respect to training design and motivation. In particular, interventions that occurred before and after dialysis resulted in a high dropout rate due to motivational reasons and lack of time, especially in adolescents. Also, individualization of training sessions, e.g., time limits, and increase or decrease of workload are necessary points to consider, when performing endurance training during dialysis [2, 9, 10]. In a mono-centric Italian study with 10 participants, it was shown that an intradialytic bicycle ergometer training of 30 min’ duration led to a significant increase of 6-min walking test performance [25].

We were able to show that a training program during dialysis for pediatric/adolescent patients on maintenance HD is doable and safe. We experienced no training-related sAEs, which would have hampered the training program. However, we experienced a loss of motivation in most of our patients over the time of the study. This was especially the case in adolescent patients. They obviously did not regard the training as a possibility to increase their quality of life, which we had acknowledged in our previous study [26]. In the latter, an obvious increase in endurance capacity was seen and patients reported to better be able to participate in normal daily life, as well as in social or sports events. In the current study, however, no effect was seen when watt-related workload was measured, or quality of life questionnaires were analyzed (see supplemental material). This may be due to the fact that only already sportive, and hence primarily motivated, patients participated in this study, which was definitively not the case in our pilot study years ago [26].

According to the literature, we had speculated that an increased muscle blood flow and greater amount of open capillary surface area in training muscles during dialysis would result in a greater flux of urea and associated toxins from tissue to the vascular compartment with subsequent removal via dialysis [14]. In the contrary, we did not find evidence that the exercise settings used for our study are inducing such a flux of urea as no increased elimination was achieved.

The small number of participants, especially in the per-protocol group, could be one of the reasons why no endpoint was reached. Only nine patients finished per-protocol in the intervention group, whereas at least 26 were expected. But also, in the ITT population, a low number of evaluable patients were achieved. Even subgroup evaluations (see supplemental material) did not find evidence for group-specific advantages. There were several reasons for the missing participants: fewer patients randomized, a significant number of patients transplanted during the trial, test protocol deviations, the long duration of the study and thus the decrease of including patients, e.g., because of competing studies, and last but not least the low motivation of the patients to train as planned. It was interesting to see a high motivation at the beginning of the training periods. Also, patients in the waiting control group or patients not yet included into the study were extremely eager to start training. Very soon, however, they complained about too much effort and too time-consuming and wanted to get back to their routine dialysis procedures. Only a minority of patients asked for further training after the study was stopped (less than 10%).

We also have to critically ask ourselves whether the intervention had not enough intensity to force an effect. Endurance-oriented exercise on the stationary ergometer, which is monitored by continuous blood pressure and heart rate measurements as used in many cardiovascular diseases in children and adolescents, has proved its efficacy in this age group of patients with chronic kidney insufficiency [10]. The growing organism has a high adaptation to aerobic performance and is well suited for endurance exercise in an aerobic state [12]. In order to develop a child-appropriate training concept, we had to consider the everyday movements of a child: they perform short, fast movements in an aerobic state, similar to an interval training [13]. This form of exercise is an attractive alternative to classic “high volume training.” In addition, it offers numerous combinations in terms of the ratio of “exercise-to-pause” duration, mean intensity, and amplitude of intensity to training pauses [14]. In addition to a smaller time investment and rapidly noticeable changes, this form of endurance exercise has demonstrated improvements in performance physiology [15]. So, we had many reasons to use the endurance program as planned for this study and we still think that it is an appropriate method to train during dialysis. We may, however, make the training more interesting and more playful, so that the patients are more interested to stay on track. We had discussed a bike training which would have been accompanied by a computer program (e.g., 30-min ride through a hilly region). However, we did not use such a program, as we thought it would have been difficult to find appropriate interventions for a pediatric patient population and it was not really accessible at the time of the study design. Nevertheless, as it was harder than expected to motivate patients to participate regularly, it might have been better to use, e.g., common motivational methods from fitness centers. Here, group training would have been an idea, which was, however, not feasible because of patient randomization timelines and facility circumstances (e.g., rooms too small). Furthermore, it is essential, but also difficult, to motivate medical staff to recruit participants and keep the dropout rate low.

Nevertheless, we have to consider that the trial had not enough power to prove neither an effect on dialysis efficacy nor any of the secondary endpoints. We can only state that it was safe to perform intradialytic bicycle ergometer training and that it did not have a negative effect on the participants. We still think that such an endurance training program would be a positive measure in patients with maintenance HD, but that it would be necessary to profoundly upgrade the circumstances. It possibly would have been better to perform a study without a control group and with a 3 months training program only. Also, for a future study, motivational aspects need to be considered more carefully. Possibly new technical innovations such as fitness bracelets and watches and the use of interactive (training games) could be helpful here. It also has to be taken into account that pediatric and adolescent patients do not want to perform sports during dialysis, but would much more appreciate adapted sports programs with their friends during their spare time.

## Conclusion

Intradialytic bicycle training is safe, but does not improve dialysis efficacy and physical fitness. However, the study can be considered underpowered, particularly because of high dropout rates. Future studies need better strategies to increase motivation and compliance and other more effective/intensive exercise measures should be evaluated.

## Supplementary information

ESM 1 (PPTX 56 kb)

ESM 2 (PPTX 760 kb)
