# Supplementary material for: Endurance-oriented training program with children and adolescents on maintenance hemodialysis to enhance dialysis efficacy—DiaSport
Source: Pediatr Nephrol. 2021 Jun 12;36(12):3923–32. doi: 10.1007/s00467-021-05114-8 (PMC8599370; doi:10.1007/s00467-021-05114-8)
Supplement: Supplementary file 1 — (PPTX 56 kb) [file 467_2021_5114_MOESM1_ESM.pptx]

## Slide 1
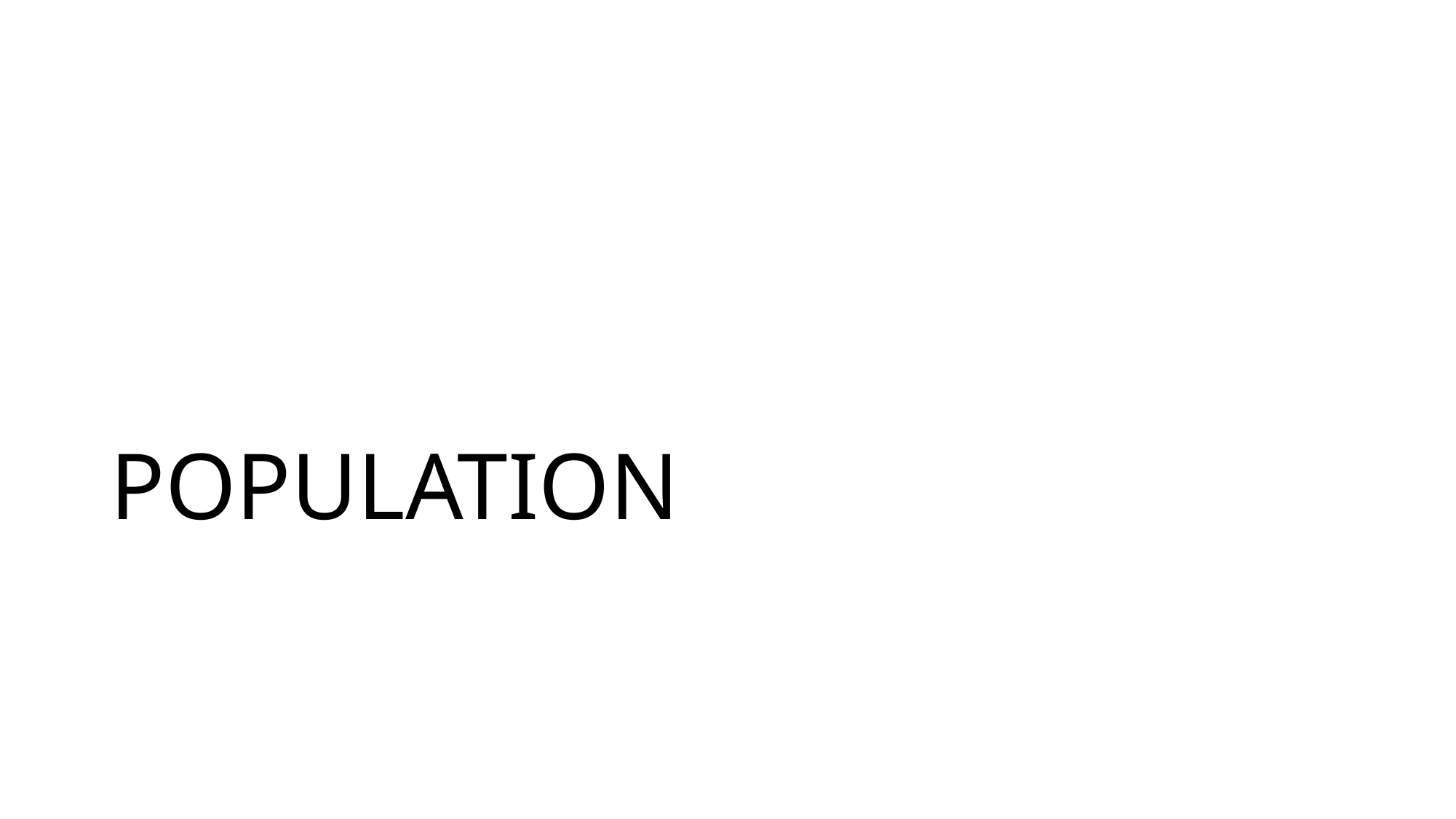

# POPULATION

## Slide 2
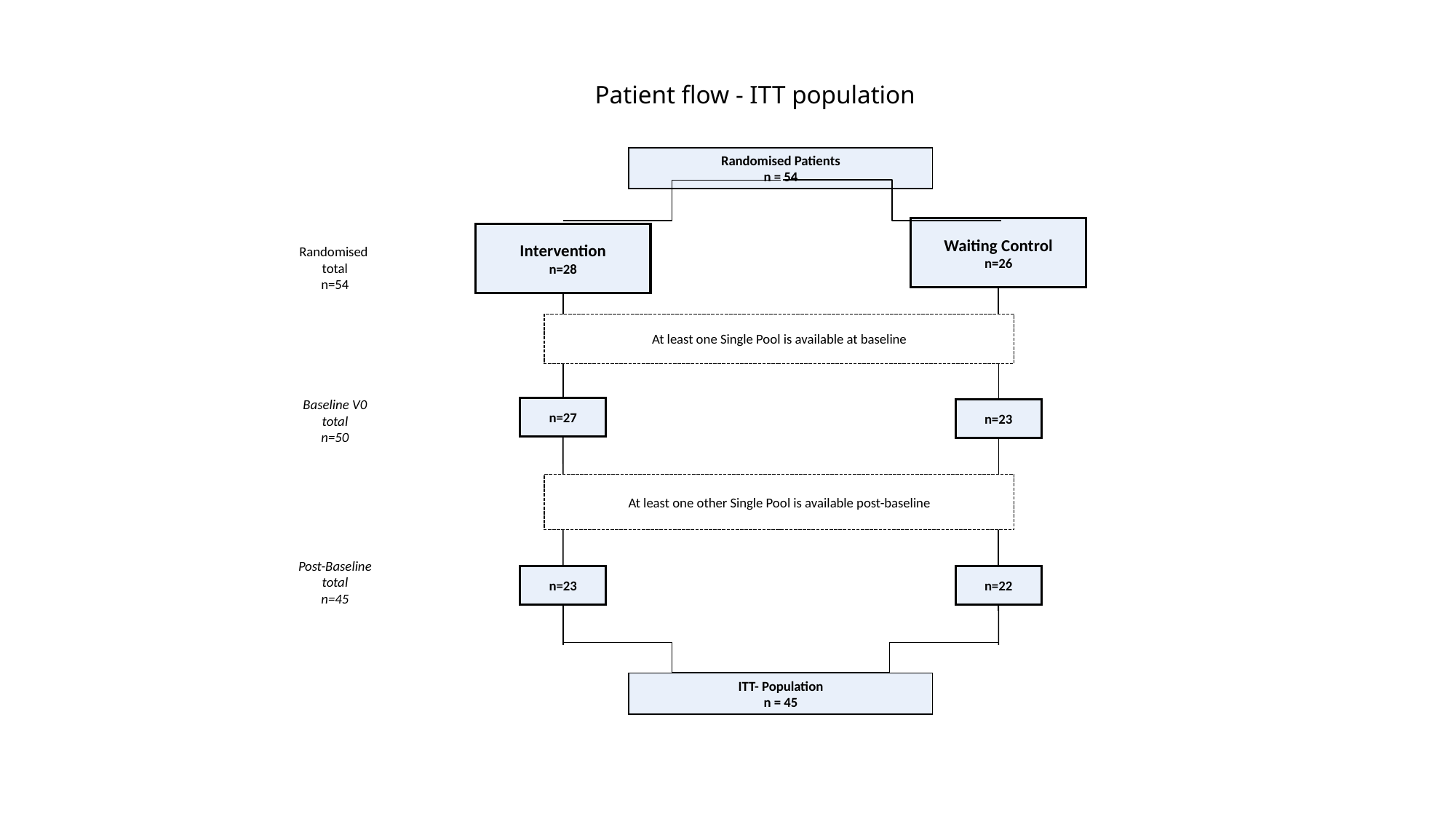

# Patient flow - ITT population
Randomised Patients
n = 54
Waiting Control
n=26
Intervention
n=28
Randomised
total
n=54
At least one Single Pool is available at baseline
Baseline V0
total
n=50
n=27
n=23
At least one other Single Pool is available post-baseline
Post-Baseline
total
n=45
n=23
n=22
ITT- Population
n = 45

## Slide 3
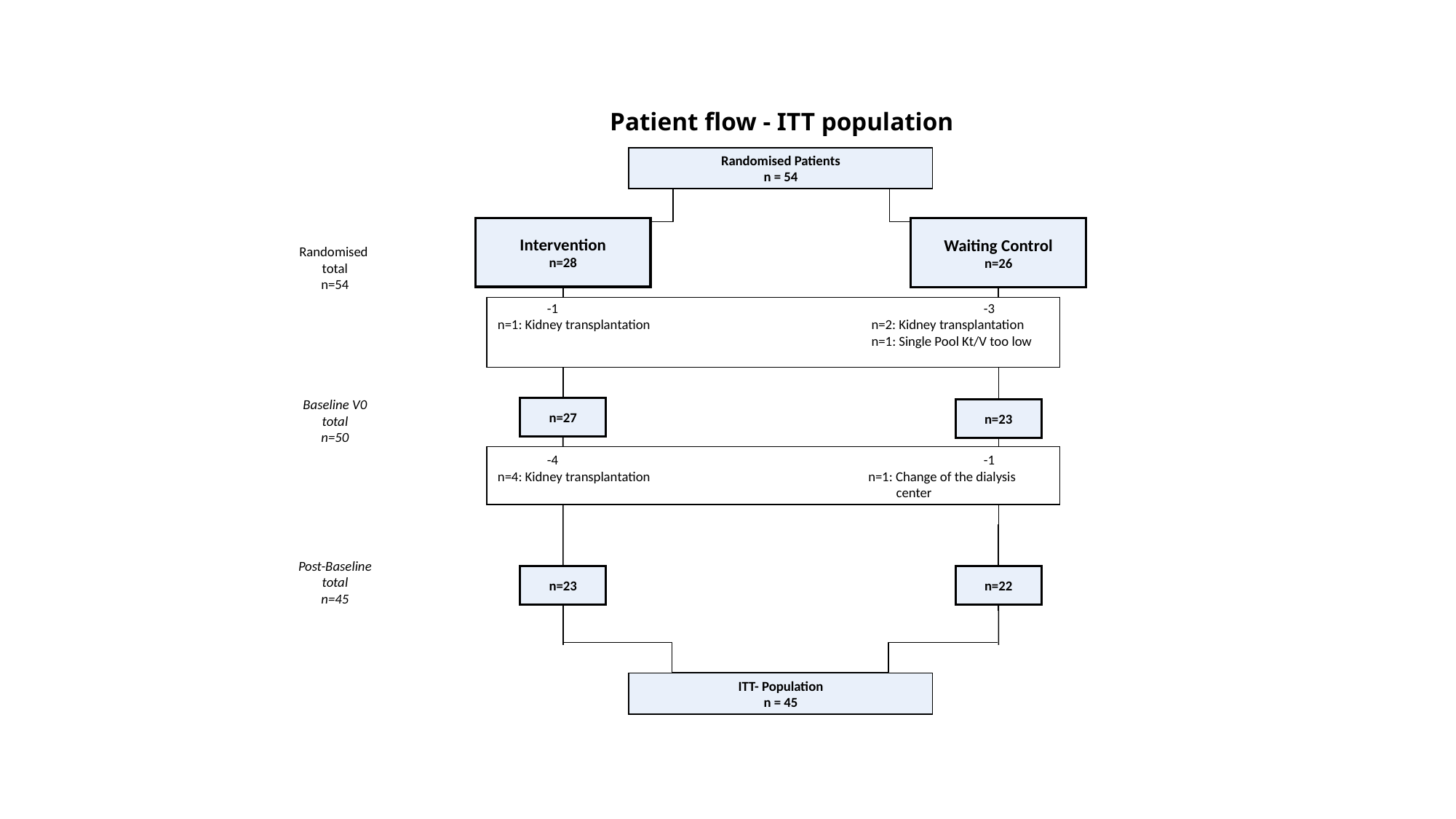

# Patient flow - ITT population
Randomised Patients
n = 54
Intervention
n=28
Waiting Control
n=26
Randomised
total
n=54
 -1	 	 	 -3
n=1: Kidney transplantation		 n=2: Kidney transplantation
			 n=1: Single Pool Kt/V too low
Baseline V0
total
n=50
n=27
n=23
 -4		 	 	 -1
n=4: Kidney transplantation		 n=1: Change of the dialysis
			 center
Post-Baseline
total
n=45
n=23
n=22
ITT- Population
n = 45

## Slide 4
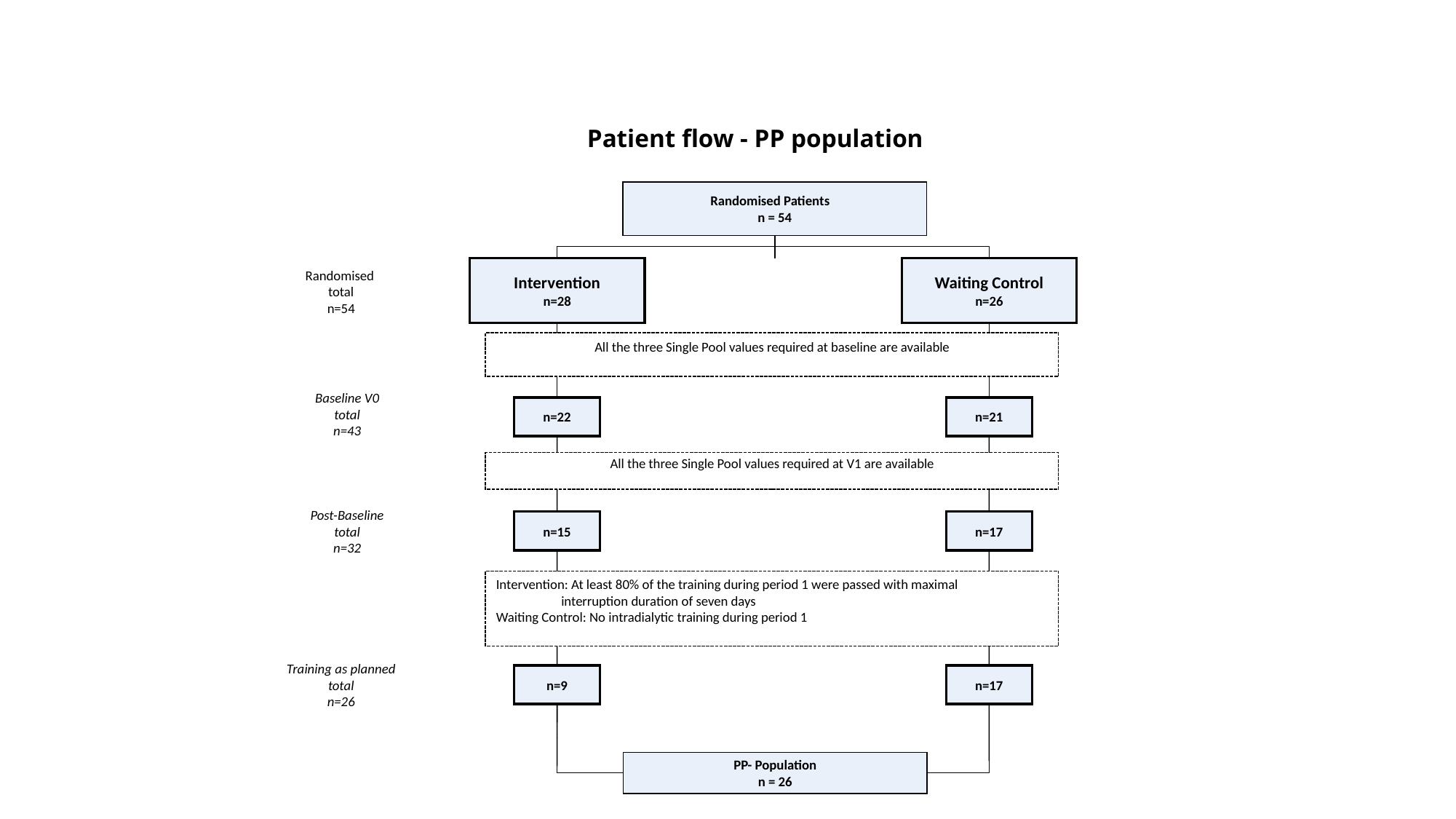

Patient flow - PP population
Randomised Patients
n = 54
Intervention
n=28
Waiting Control
n=26
Randomised
total
n=54
All the three Single Pool values required at baseline are available
Baseline V0
total
n=43
n=22
n=21
All the three Single Pool values required at V1 are available
Post-Baseline
total
n=32
n=15
n=17
Intervention: At least 80% of the training during period 1 were passed with maximal
 interruption duration of seven days
Waiting Control: No intradialytic training during period 1
Training as planned
total
n=26
n=9
n=17
PP- Population
n = 26

## Slide 5
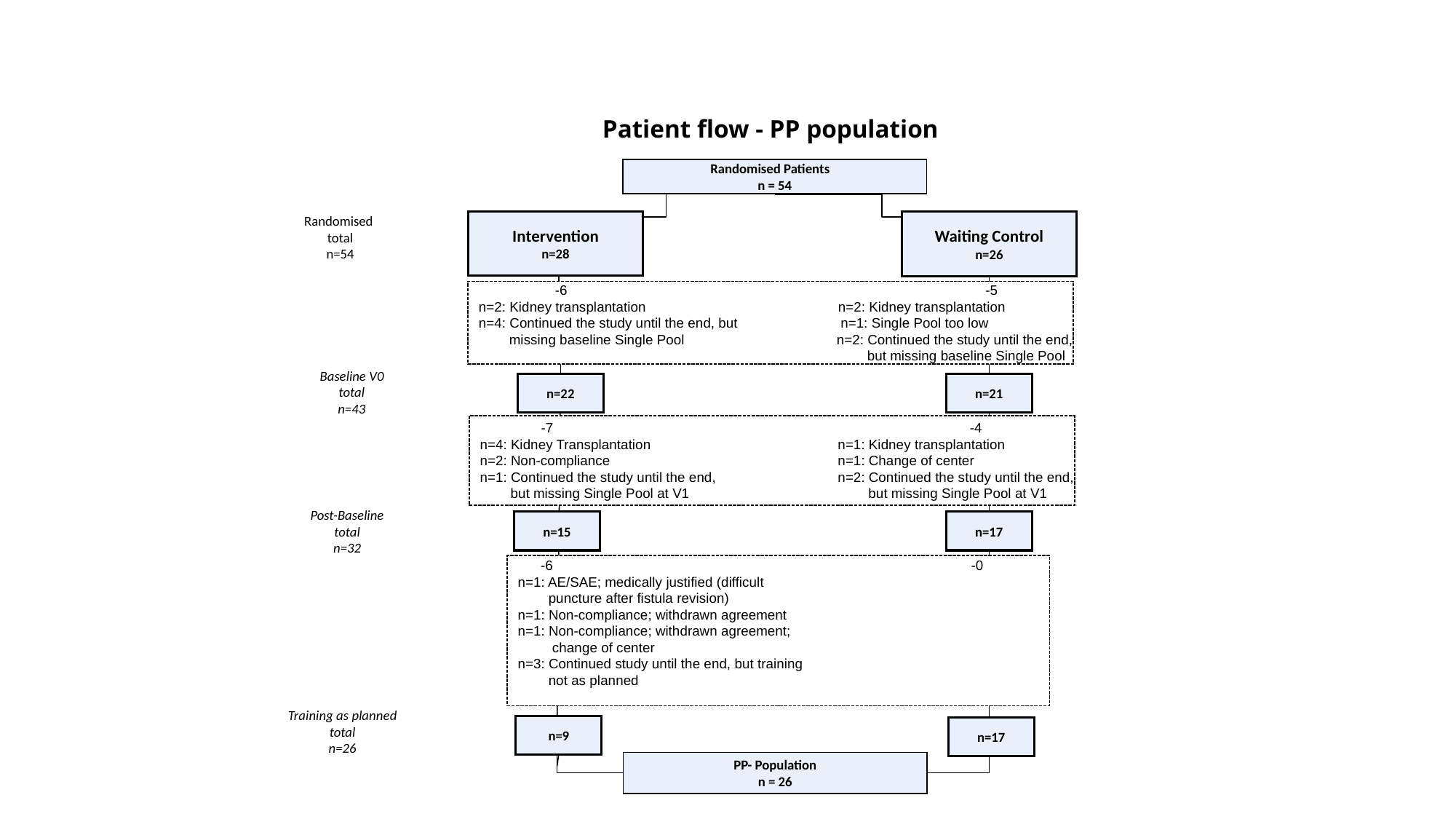

Patient flow - PP population
Randomised Patients
n = 54
Intervention
n=28
Randomised
total
n=54
Waiting Control
n=26
 -6	 	 -5
n=2: Kidney transplantation	 n=2: Kidney transplantation
n=4: Continued the study until the end, but n=1: Single Pool too low
 missing baseline Single Pool 		 n=2: Continued the study until the end,
			 but missing baseline Single Pool
Baseline V0
total
n=43
n=22
n=21
 -7			 	 -4
n=4: Kidney Transplantation		 n=1: Kidney transplantation
n=2: Non-compliance		 n=1: Change of center
n=1: Continued the study until the end, 	 n=2: Continued the study until the end,
 but missing Single Pool at V1		 but missing Single Pool at V1
Post-Baseline
total
n=32
n=15
n=17
 -6	 		 -0
n=1: AE/SAE; medically justified (difficult
 puncture after fistula revision)
n=1: Non-compliance; withdrawn agreement
n=1: Non-compliance; withdrawn agreement;
 change of center
n=3: Continued study until the end, but training
 not as planned
Training as planned
total
n=26
n=9
n=17
PP- Population
n = 26

## Slide 6
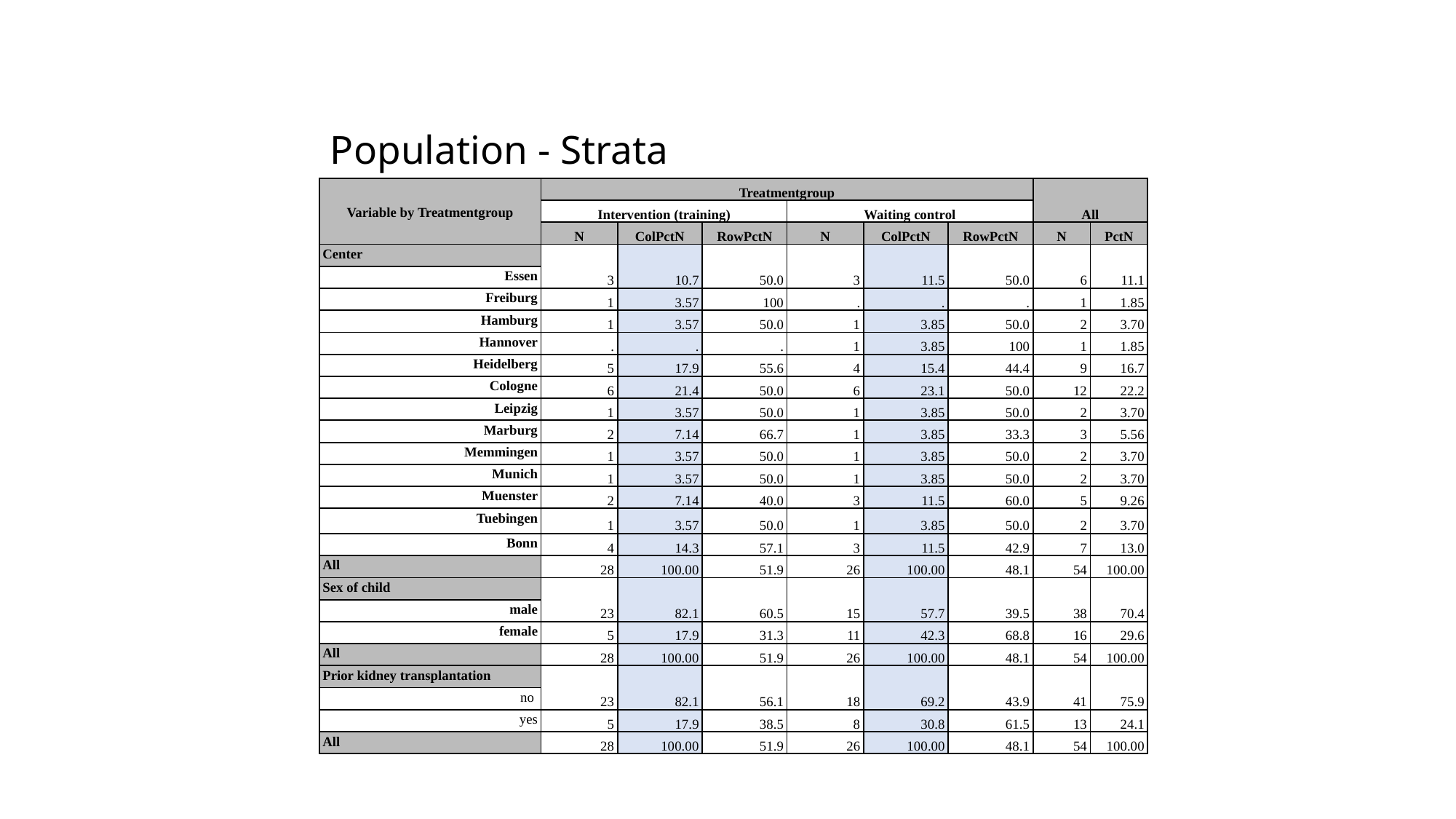

# Population - Strata
| Variable by Treatmentgroup | Treatmentgroup | | | | | | All | |
| --- | --- | --- | --- | --- | --- | --- | --- | --- |
| | Intervention (training) | | | Waiting control | | | | |
| | N | ColPctN | RowPctN | N | ColPctN | RowPctN | N | PctN |
| Center | 3 | 10.7 | 50.0 | 3 | 11.5 | 50.0 | 6 | 11.1 |
| Essen | | | | | | | | |
| Freiburg | 1 | 3.57 | 100 | . | . | . | 1 | 1.85 |
| Hamburg | 1 | 3.57 | 50.0 | 1 | 3.85 | 50.0 | 2 | 3.70 |
| Hannover | . | . | . | 1 | 3.85 | 100 | 1 | 1.85 |
| Heidelberg | 5 | 17.9 | 55.6 | 4 | 15.4 | 44.4 | 9 | 16.7 |
| Cologne | 6 | 21.4 | 50.0 | 6 | 23.1 | 50.0 | 12 | 22.2 |
| Leipzig | 1 | 3.57 | 50.0 | 1 | 3.85 | 50.0 | 2 | 3.70 |
| Marburg | 2 | 7.14 | 66.7 | 1 | 3.85 | 33.3 | 3 | 5.56 |
| Memmingen | 1 | 3.57 | 50.0 | 1 | 3.85 | 50.0 | 2 | 3.70 |
| Munich | 1 | 3.57 | 50.0 | 1 | 3.85 | 50.0 | 2 | 3.70 |
| Muenster | 2 | 7.14 | 40.0 | 3 | 11.5 | 60.0 | 5 | 9.26 |
| Tuebingen | 1 | 3.57 | 50.0 | 1 | 3.85 | 50.0 | 2 | 3.70 |
| Bonn | 4 | 14.3 | 57.1 | 3 | 11.5 | 42.9 | 7 | 13.0 |
| All | 28 | 100.00 | 51.9 | 26 | 100.00 | 48.1 | 54 | 100.00 |
| Sex of child | 23 | 82.1 | 60.5 | 15 | 57.7 | 39.5 | 38 | 70.4 |
| male | | | | | | | | |
| female | 5 | 17.9 | 31.3 | 11 | 42.3 | 68.8 | 16 | 29.6 |
| All | 28 | 100.00 | 51.9 | 26 | 100.00 | 48.1 | 54 | 100.00 |
| Prior kidney transplantation | 23 | 82.1 | 56.1 | 18 | 69.2 | 43.9 | 41 | 75.9 |
| no | | | | | | | | |
| yes | 5 | 17.9 | 38.5 | 8 | 30.8 | 61.5 | 13 | 24.1 |
| All | 28 | 100.00 | 51.9 | 26 | 100.00 | 48.1 | 54 | 100.00 |

## Slide 7
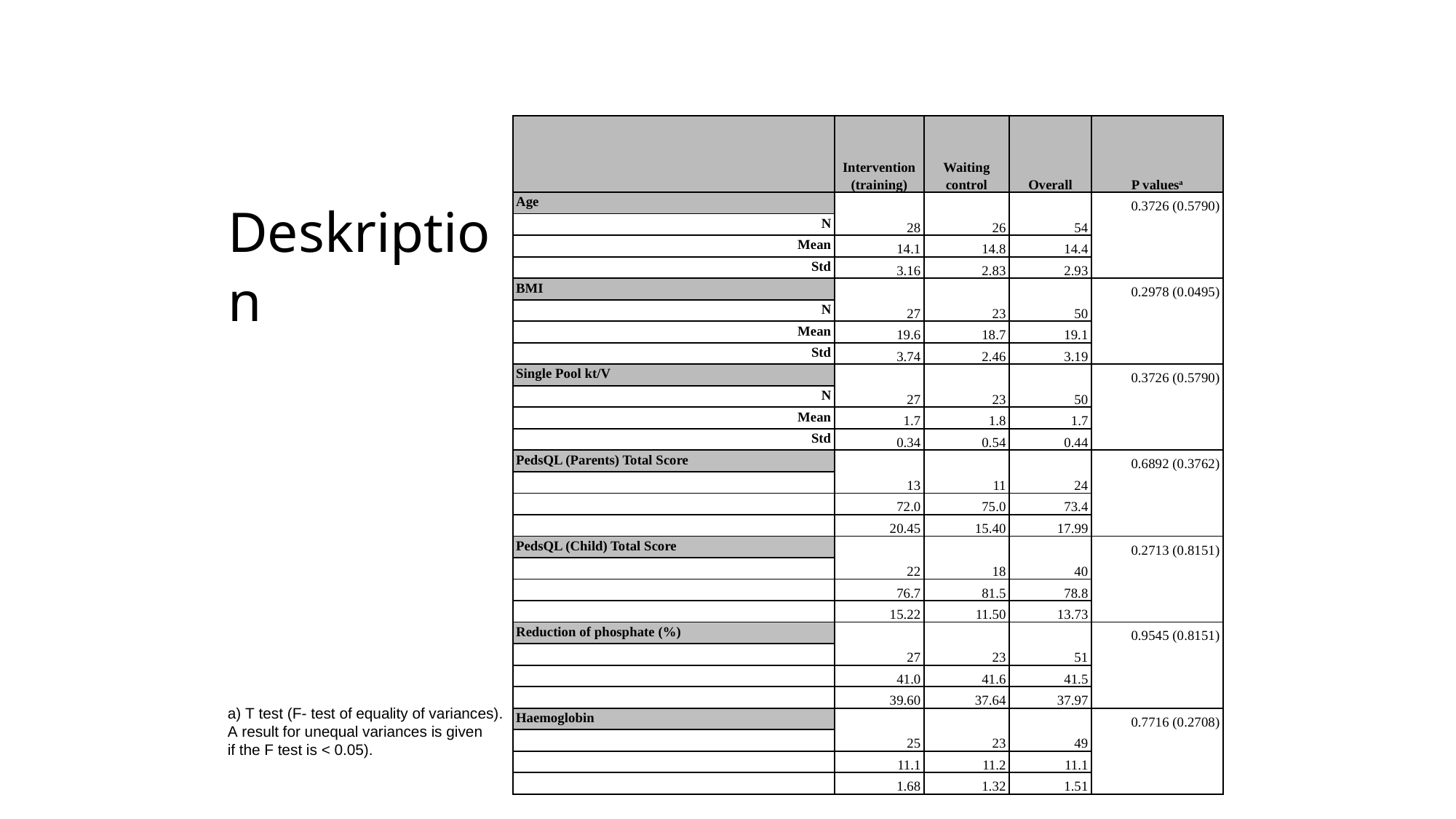

# Deskription
| | Intervention (training) | Waiting control | Overall | P valuesa |
| --- | --- | --- | --- | --- |
| Age | 28 | 26 | 54 | 0.3726 (0.5790) |
| N | | | | |
| Mean | 14.1 | 14.8 | 14.4 | |
| Std | 3.16 | 2.83 | 2.93 | |
| BMI | 27 | 23 | 50 | 0.2978 (0.0495) |
| N | | | | |
| Mean | 19.6 | 18.7 | 19.1 | |
| Std | 3.74 | 2.46 | 3.19 | |
| Single Pool kt/V | 27 | 23 | 50 | 0.3726 (0.5790) |
| N | | | | |
| Mean | 1.7 | 1.8 | 1.7 | |
| Std | 0.34 | 0.54 | 0.44 | |
| PedsQL (Parents) Total Score | 13 | 11 | 24 | 0.6892 (0.3762) |
| | | | | |
| | 72.0 | 75.0 | 73.4 | |
| | 20.45 | 15.40 | 17.99 | |
| PedsQL (Child) Total Score | 22 | 18 | 40 | 0.2713 (0.8151) |
| | | | | |
| | 76.7 | 81.5 | 78.8 | |
| | 15.22 | 11.50 | 13.73 | |
| Reduction of phosphate (%) | 27 | 23 | 51 | 0.9545 (0.8151) |
| | | | | |
| | 41.0 | 41.6 | 41.5 | |
| | 39.60 | 37.64 | 37.97 | |
| Haemoglobin | 25 | 23 | 49 | 0.7716 (0.2708) |
| | | | | |
| | 11.1 | 11.2 | 11.1 | |
| | 1.68 | 1.32 | 1.51 | |
a) T test (F- test of equality of variances).
A result for unequal variances is given
if the F test is < 0.05).
