# Supplementary material for: Endurance-oriented training program with children and adolescents on maintenance hemodialysis to enhance dialysis efficacy—DiaSport
Source: Pediatr Nephrol. 2021 Jun 12;36(12):3923–32. doi: 10.1007/s00467-021-05114-8 (PMC8599370; doi:10.1007/s00467-021-05114-8)
Supplement: Supplementary file 2 — (PPTX 760 kb) [file 467_2021_5114_MOESM2_ESM.pptx]

## Slide 1
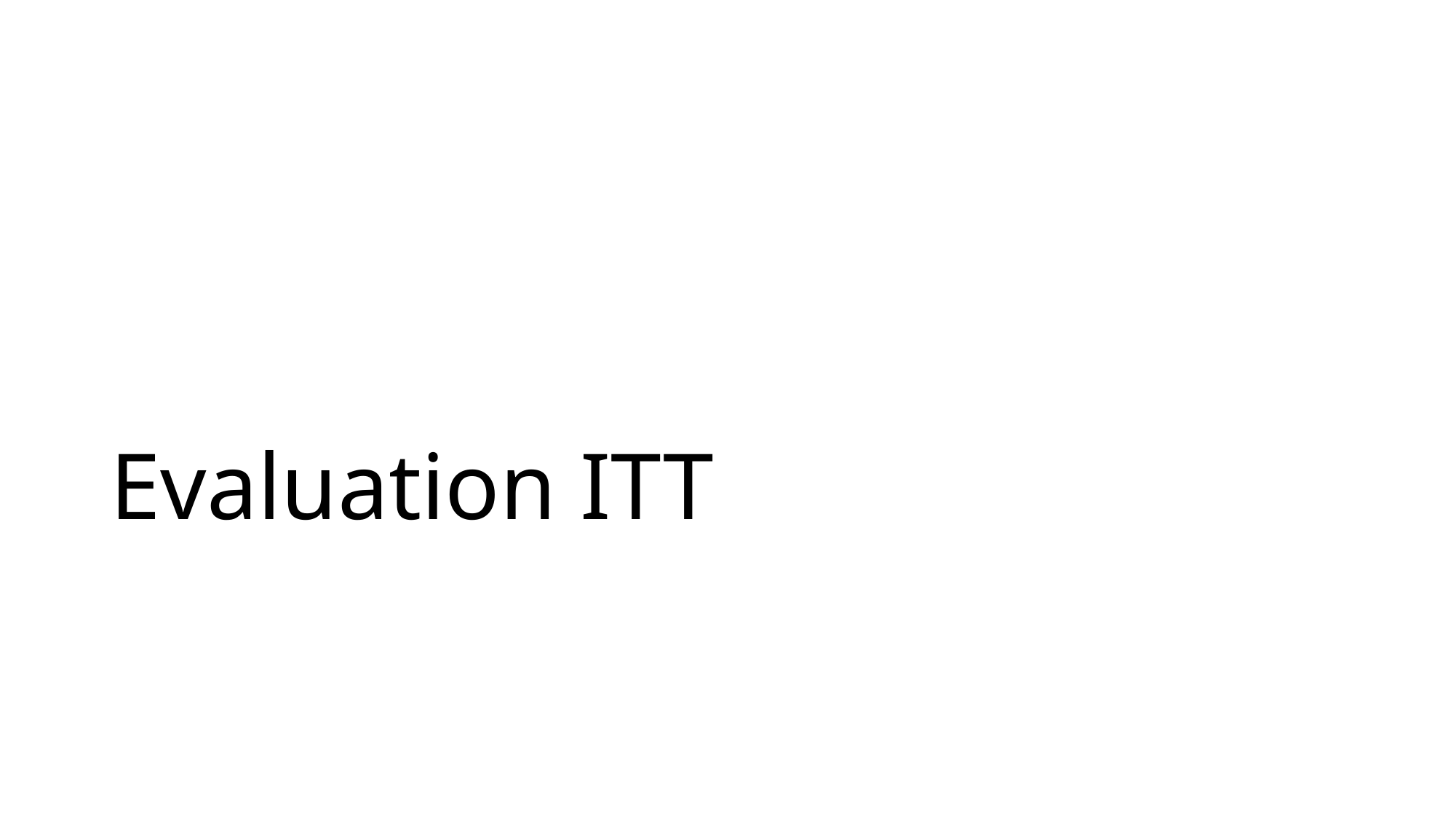

# Evaluation ITT

## Slide 2
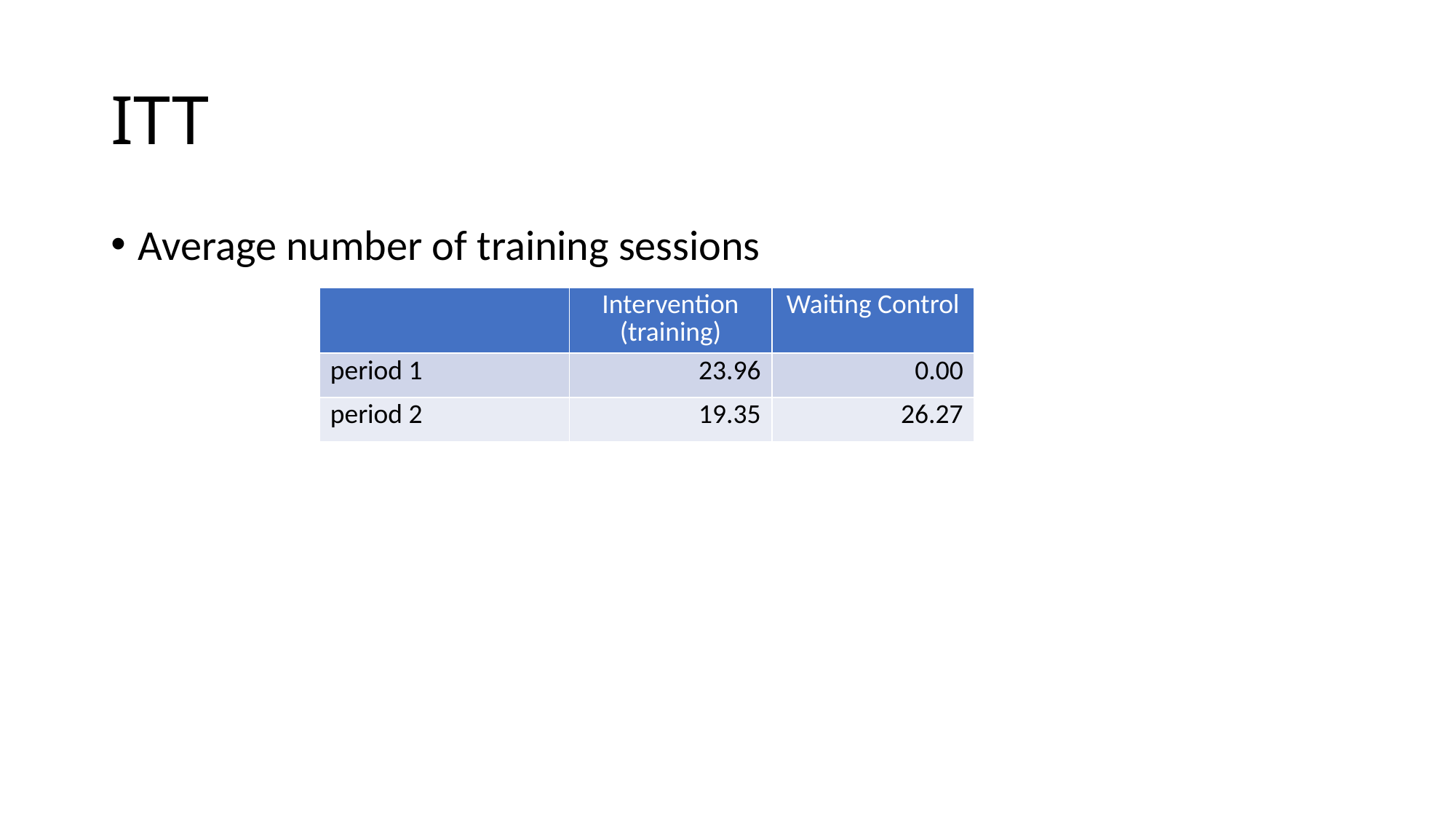

# ITT
Average number of training sessions
| | Intervention (training) | Waiting Control |
| --- | --- | --- |
| period 1 | 23.96 | 0.00 |
| period 2 | 19.35 | 26.27 |

## Slide 3
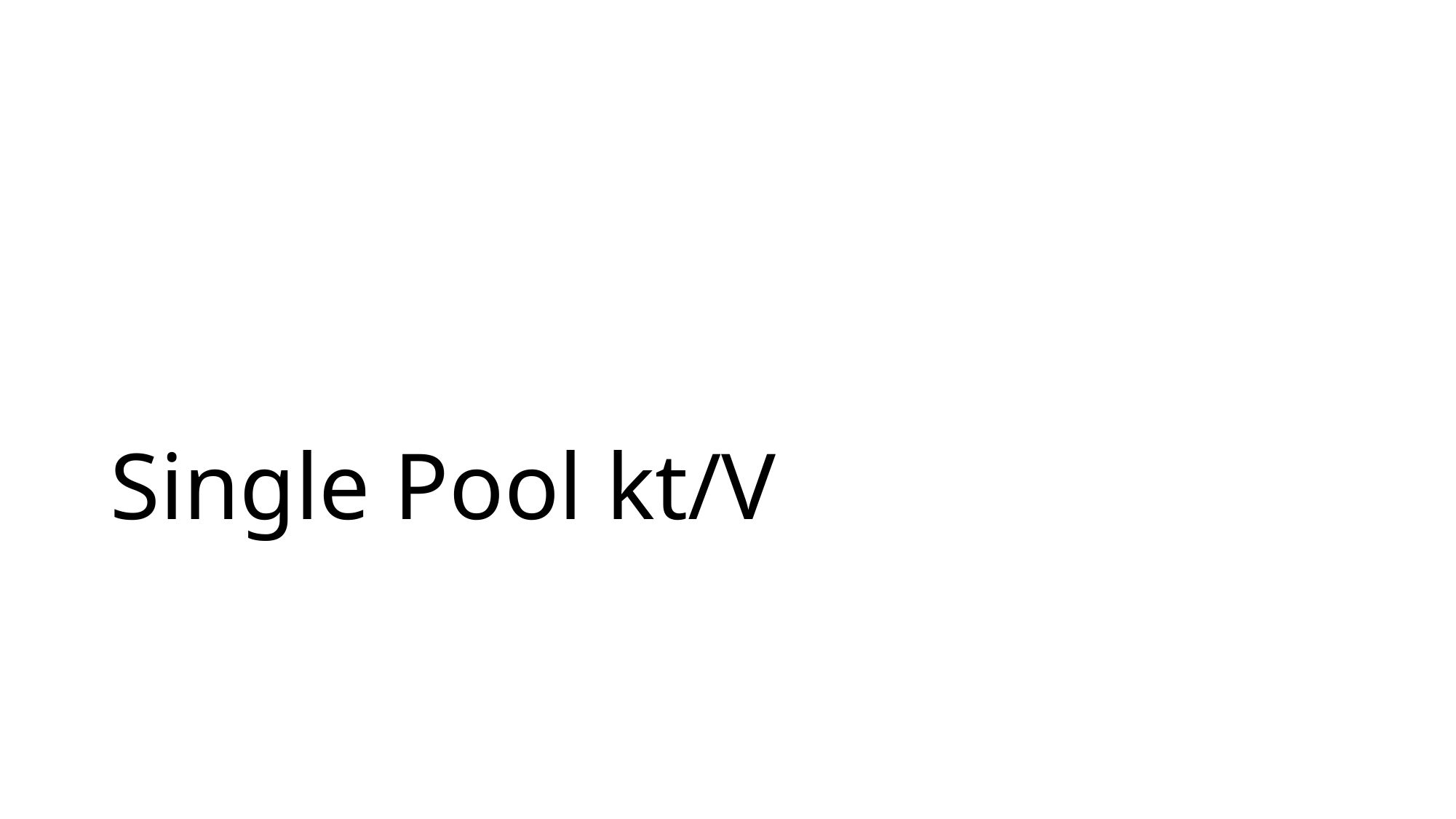

# Single Pool kt/V

## Slide 4
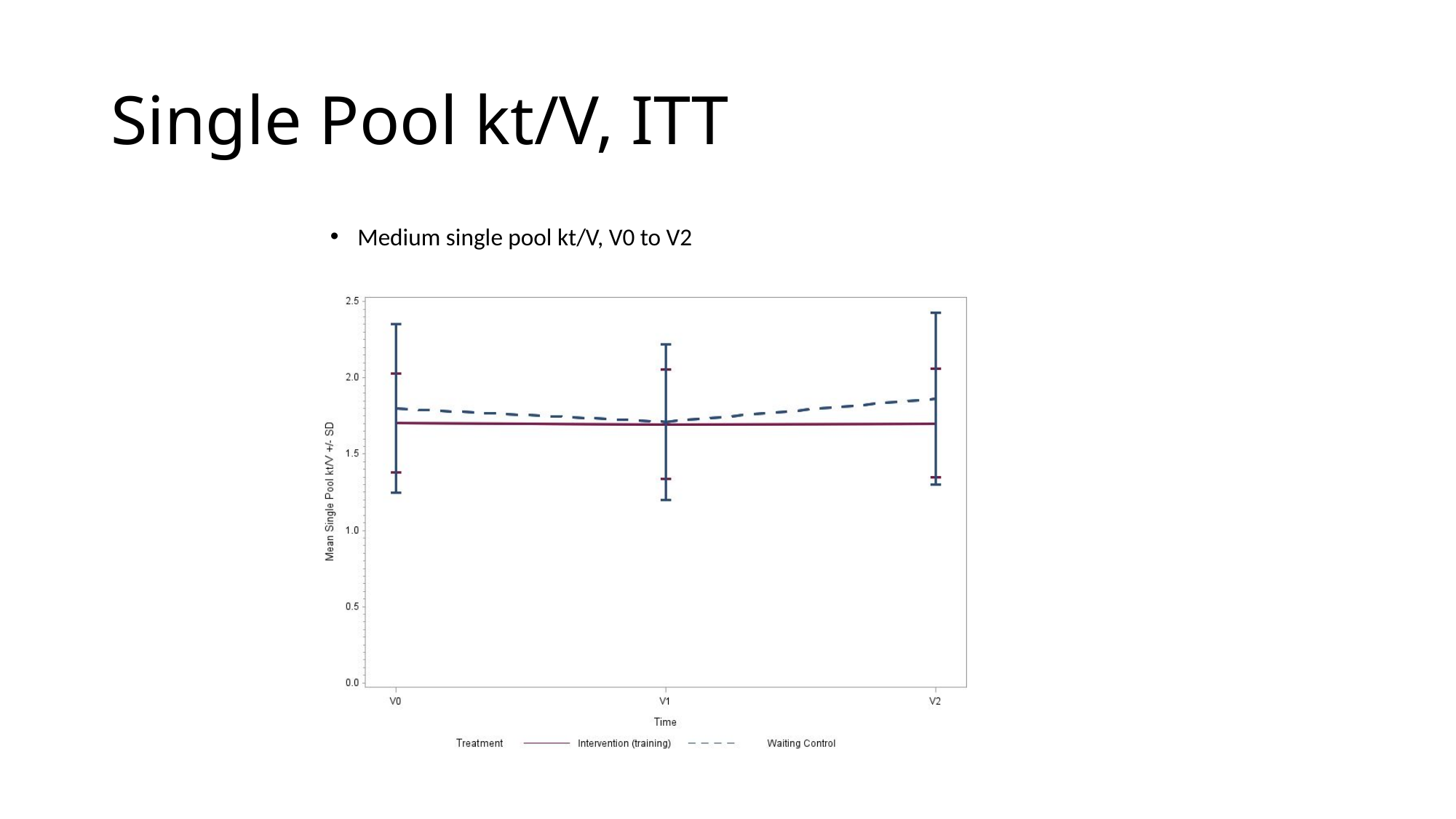

# Single Pool kt/V, ITT
Medium single pool kt/V, V0 to V2

## Slide 5
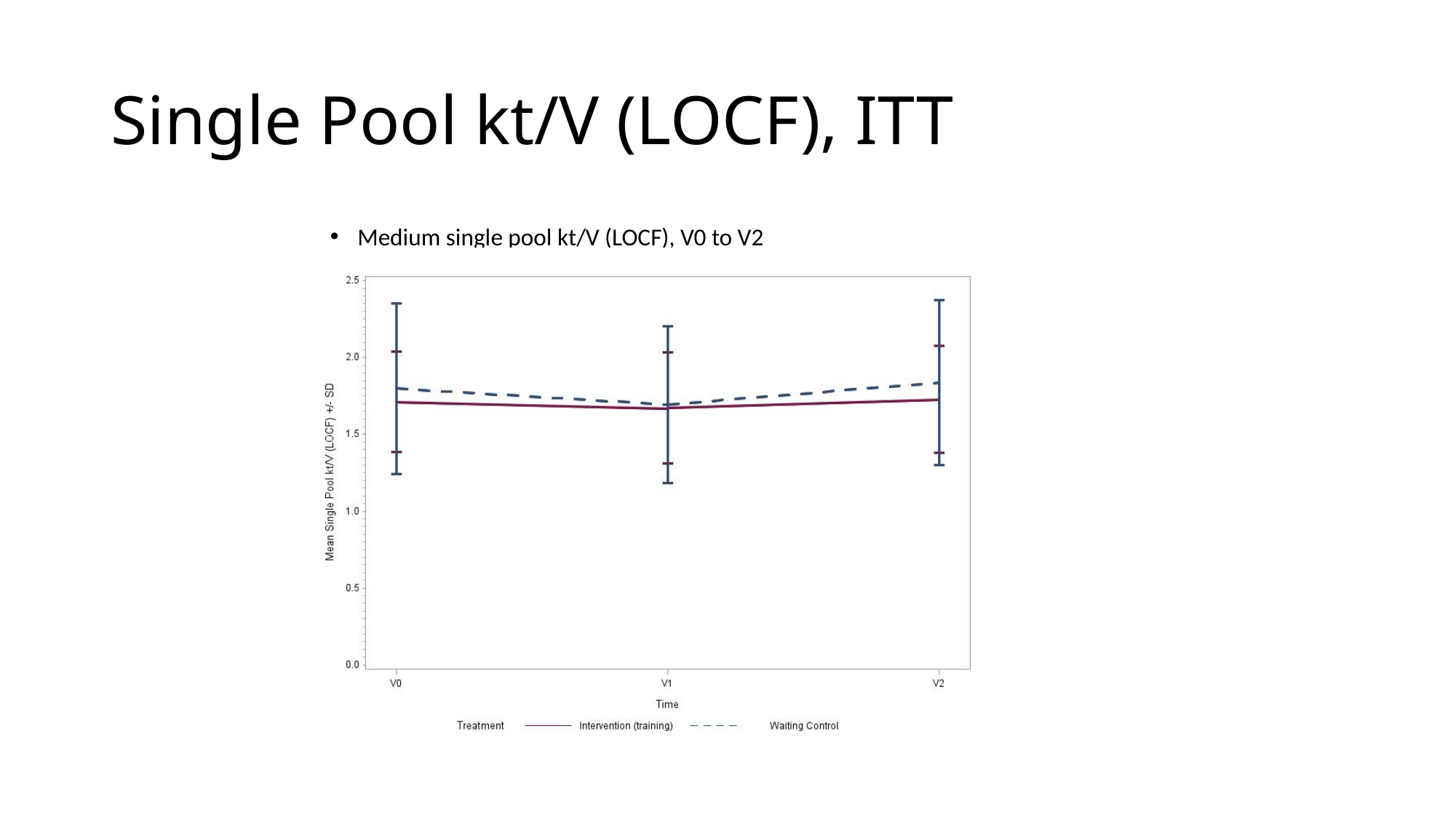

# Single Pool kt/V (LOCF), ITT
Medium single pool kt/V (LOCF), V0 to V2

## Slide 6
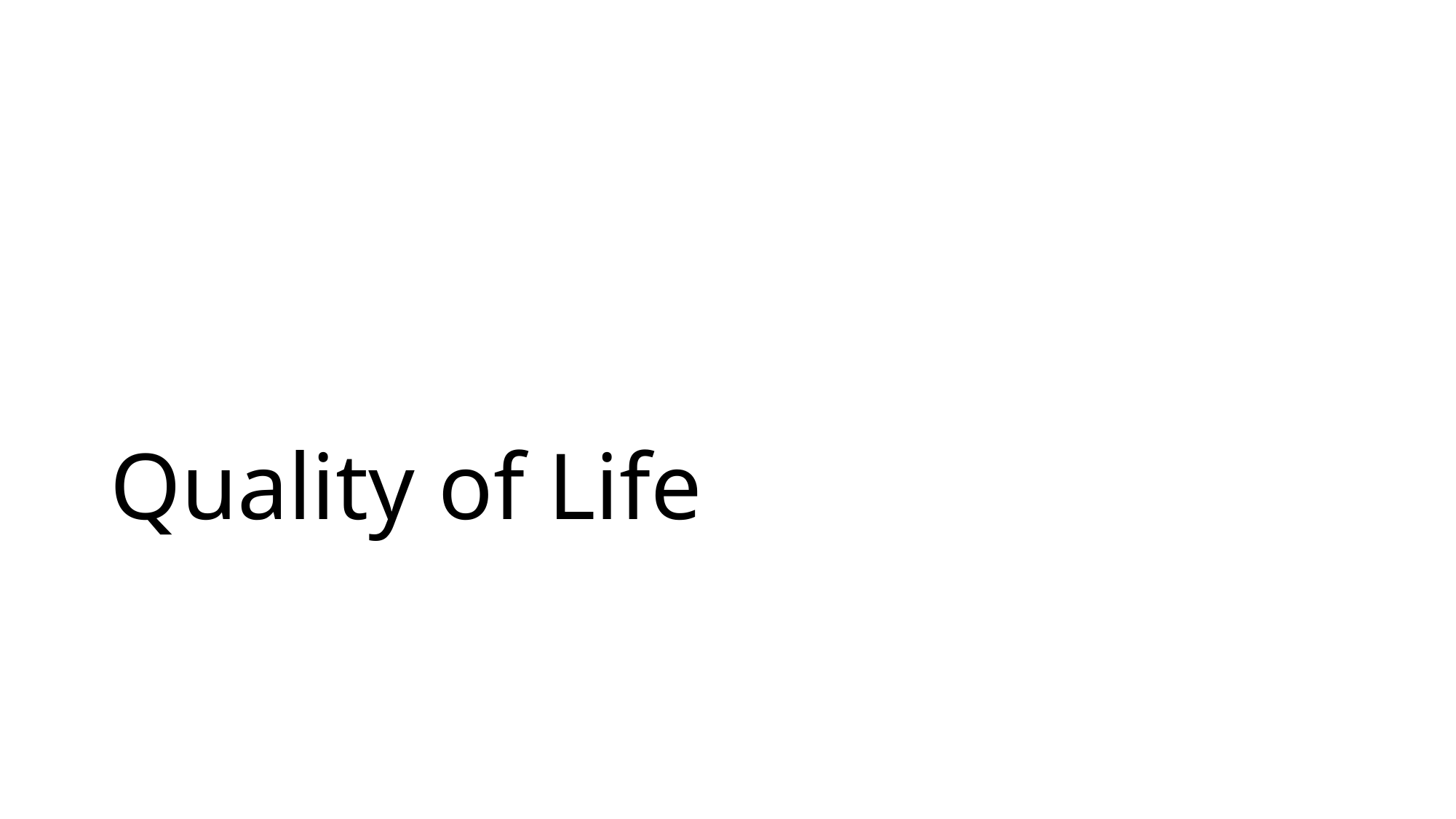

# Quality of Life

## Slide 7
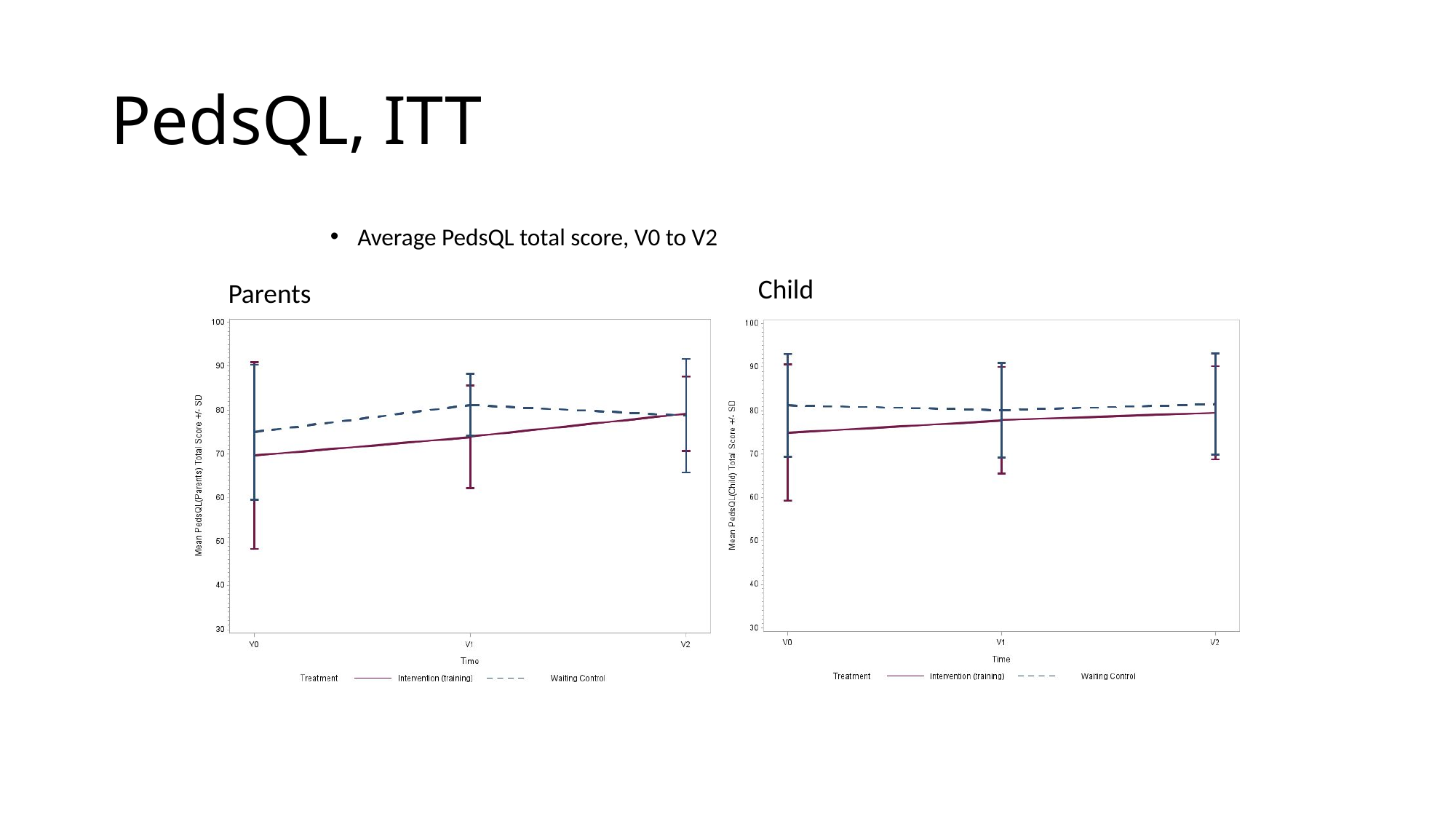

# PedsQL, ITT
Average PedsQL total score, V0 to V2
Child
Parents

## Slide 8
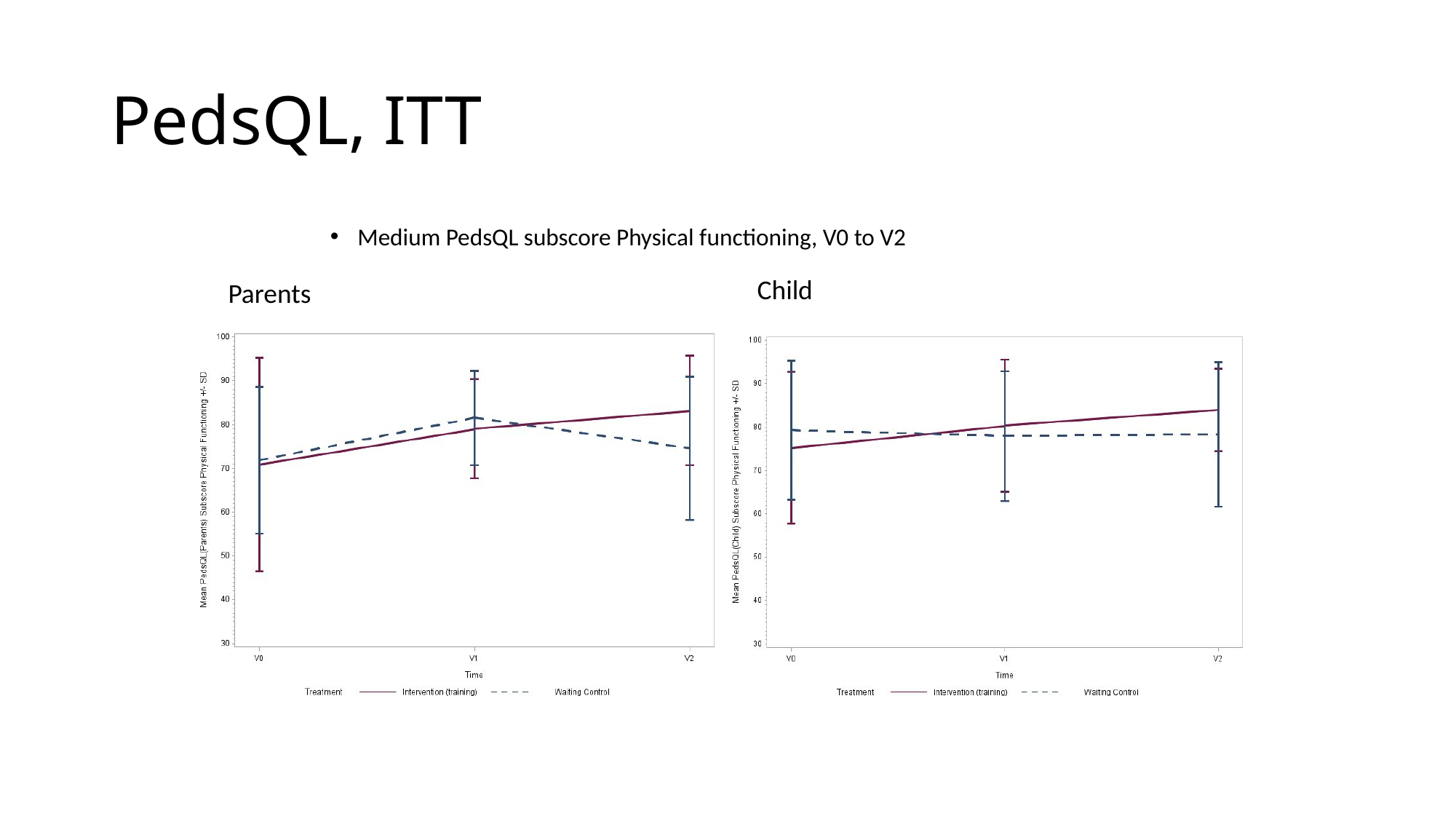

# PedsQL, ITT
Medium PedsQL subscore Physical functioning, V0 to V2
Child
Parents

## Slide 9
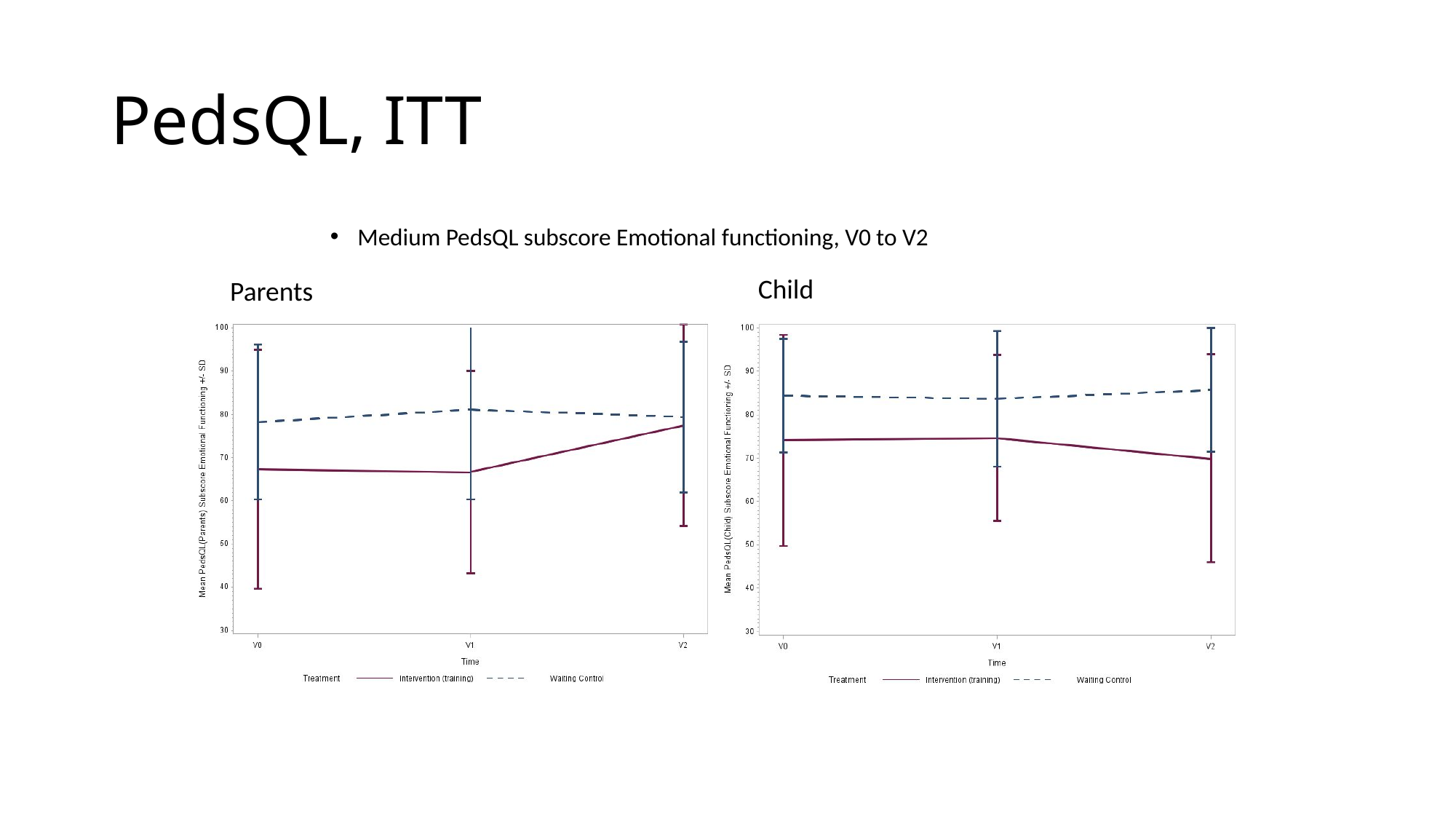

# PedsQL, ITT
Medium PedsQL subscore Emotional functioning, V0 to V2
Child
Parents

## Slide 10
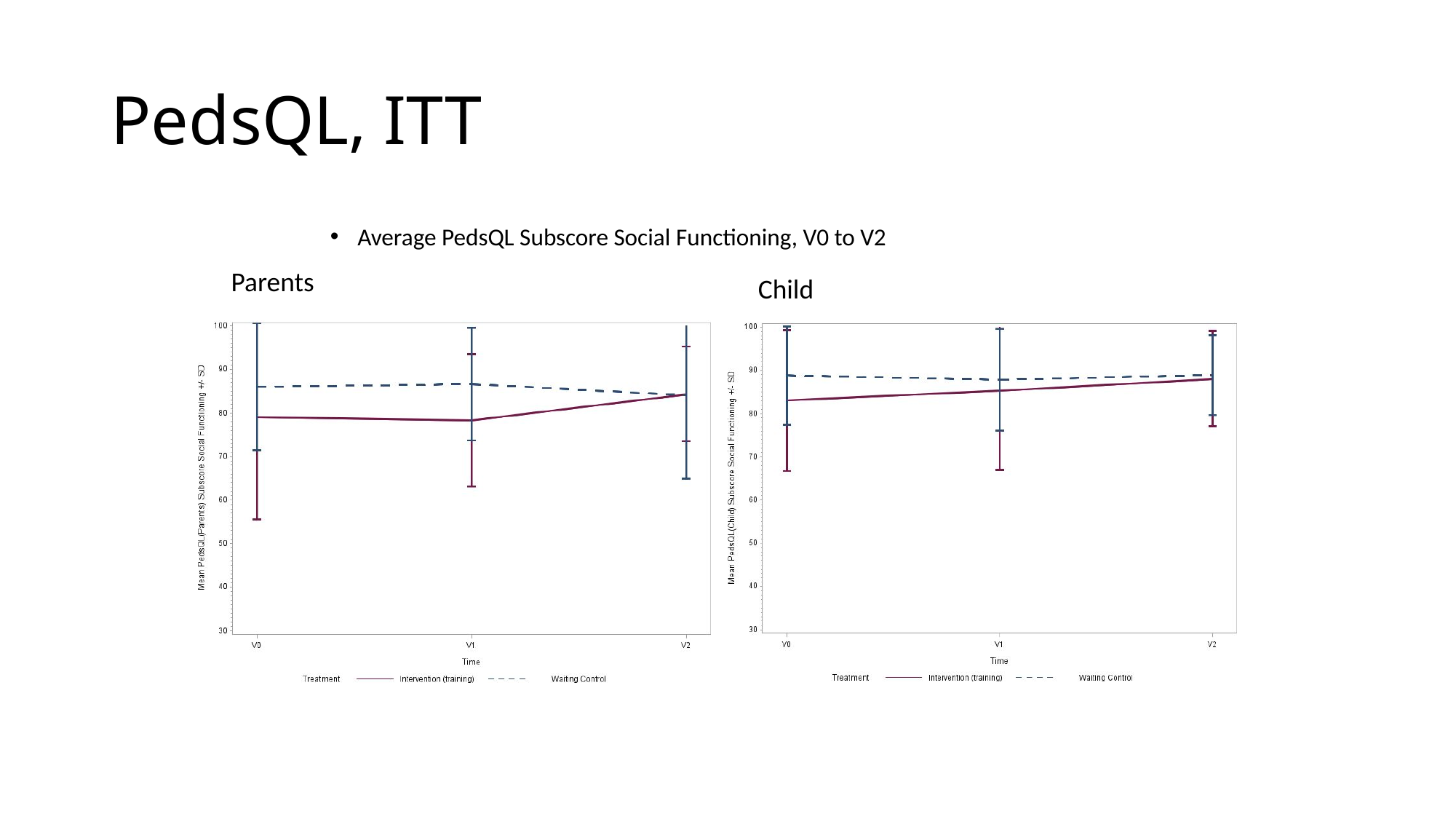

# PedsQL, ITT
Average PedsQL Subscore Social Functioning, V0 to V2
Parents
Child

## Slide 11
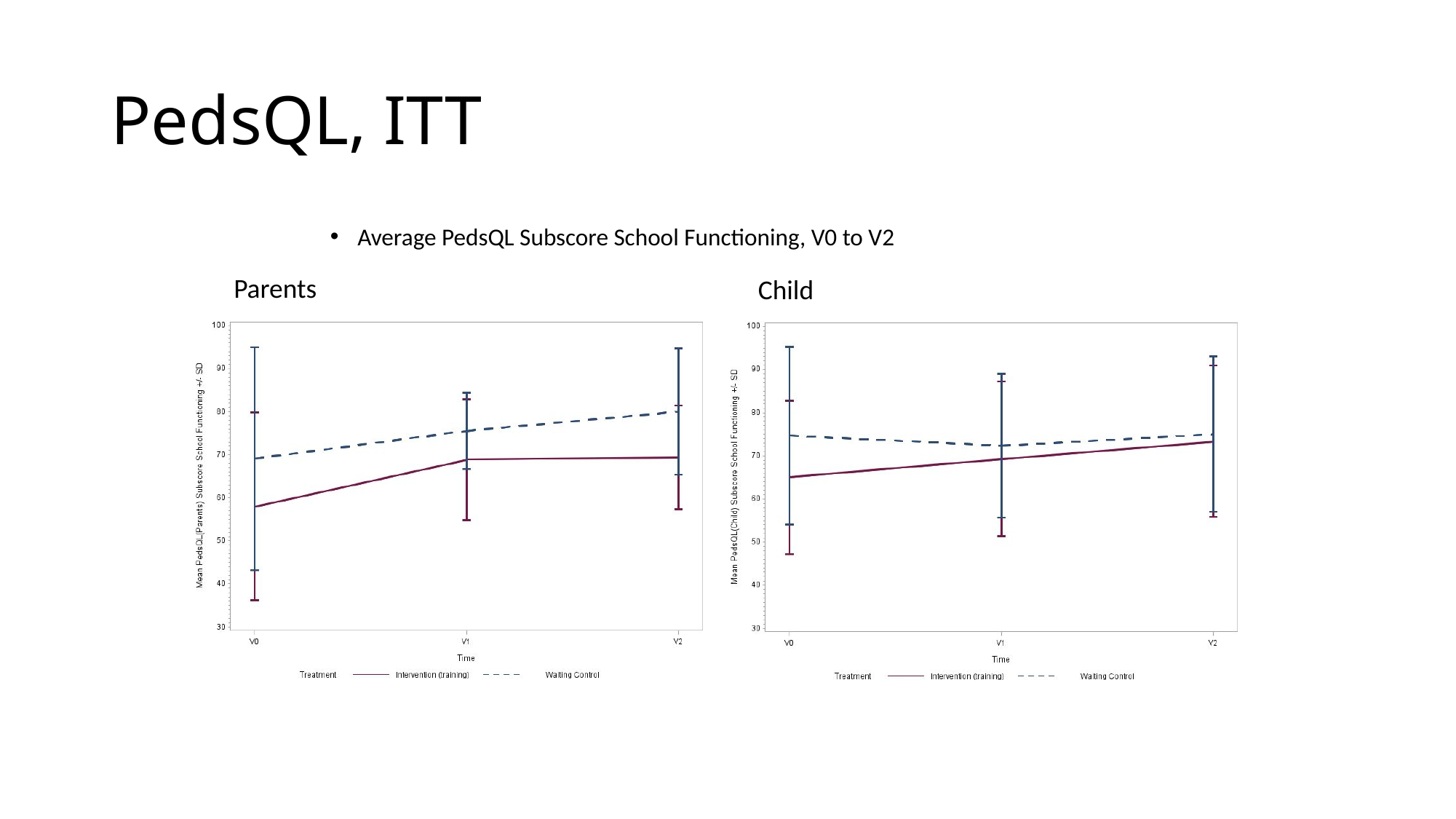

# PedsQL, ITT
Average PedsQL Subscore School Functioning, V0 to V2
Parents
Child

## Slide 12
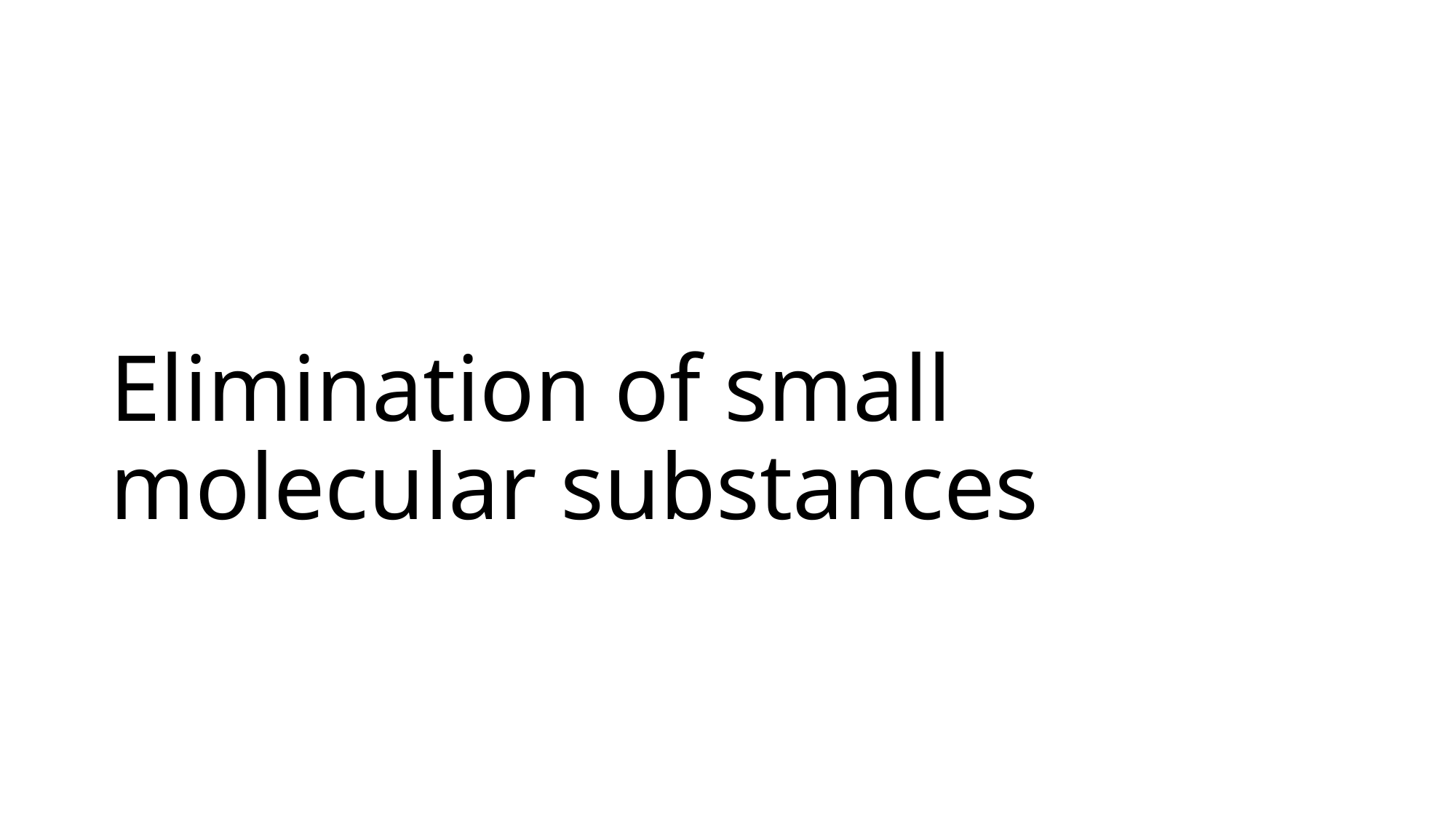

# Elimination of small molecular substances

## Slide 13
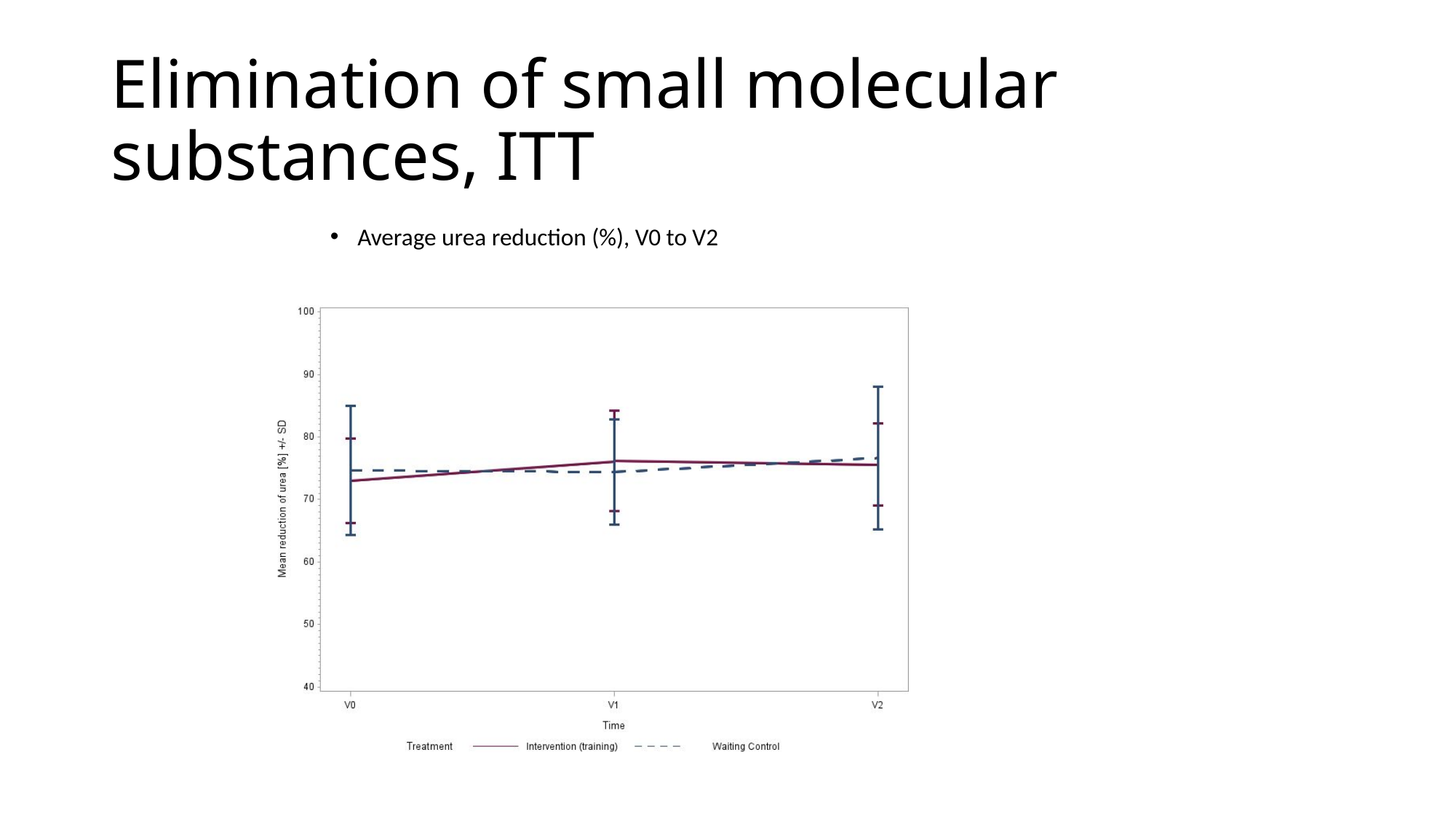

# Elimination of small molecular substances, ITT
Average urea reduction (%), V0 to V2

## Slide 14
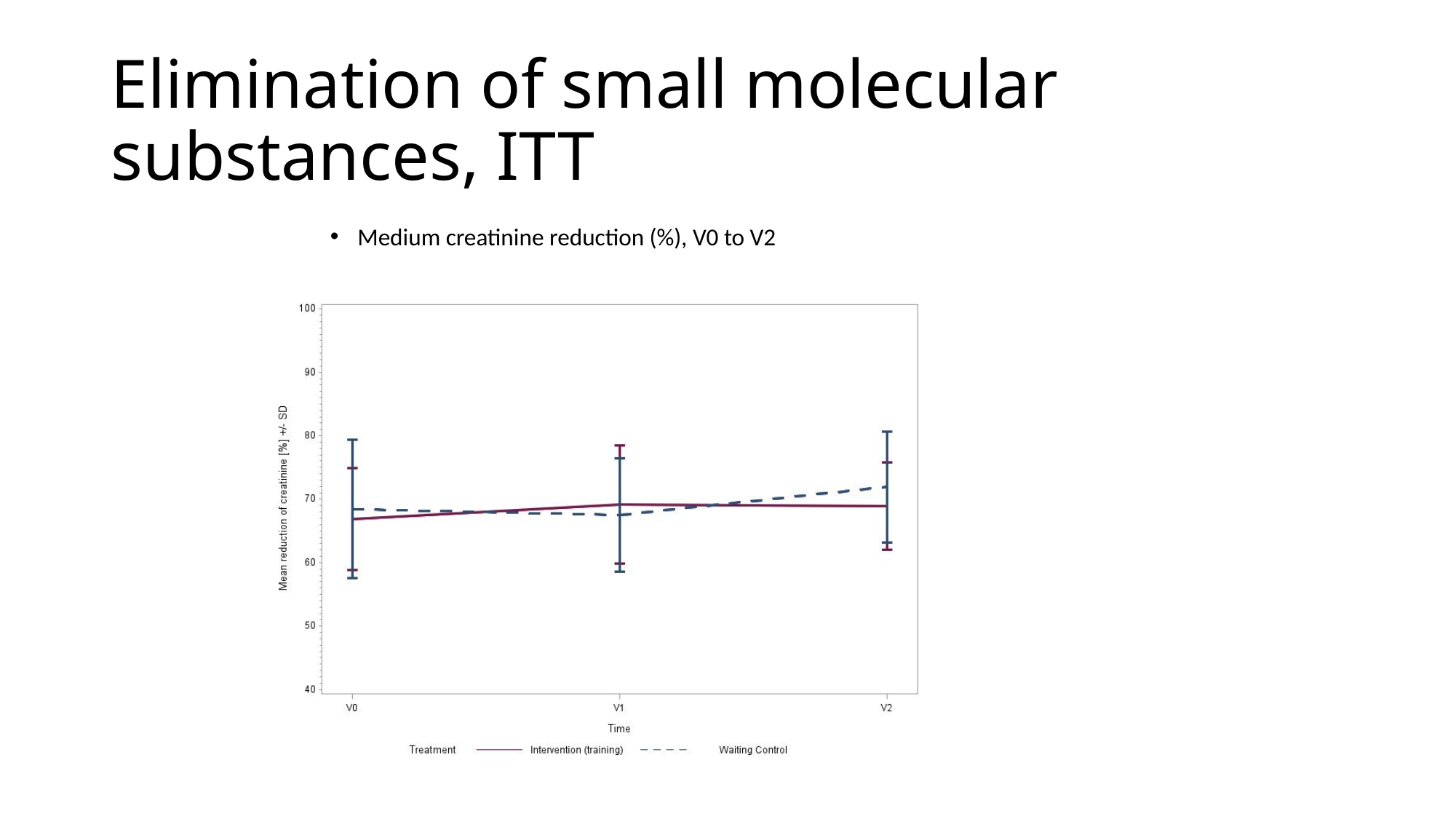

# Elimination of small molecular substances, ITT
Medium creatinine reduction (%), V0 to V2

## Slide 15
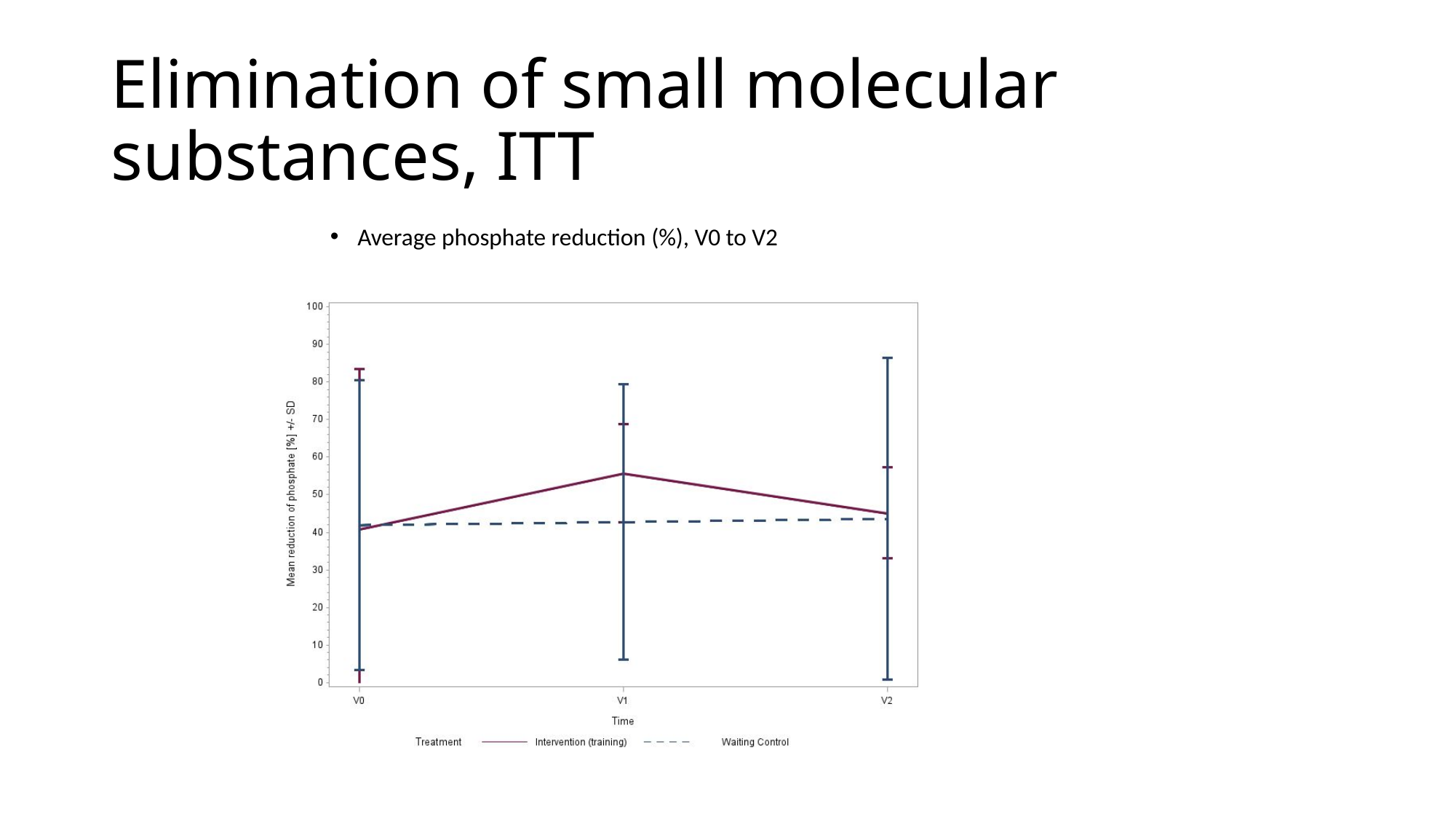

# Elimination of small molecular substances, ITT
Average phosphate reduction (%), V0 to V2

## Slide 16
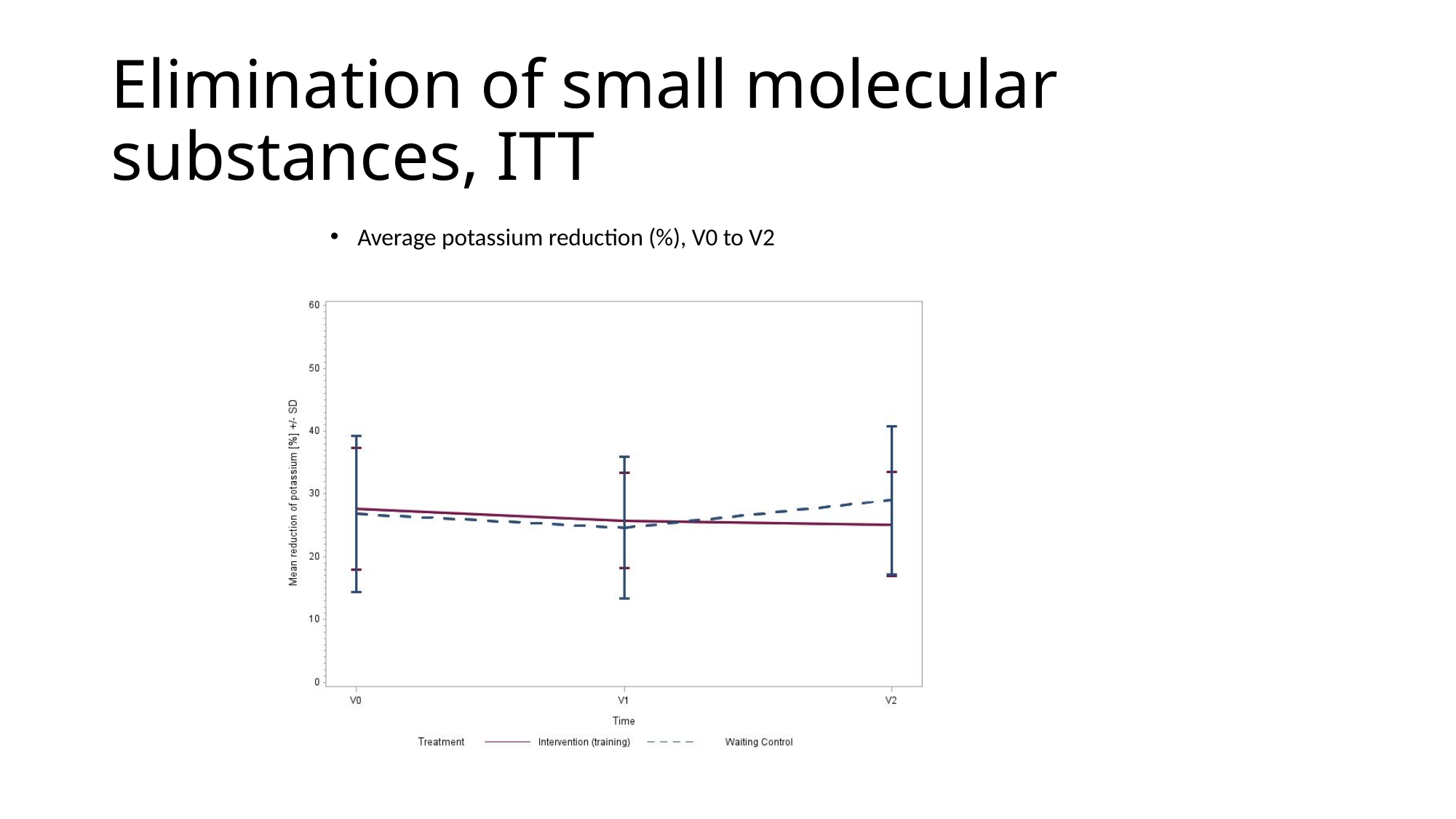

# Elimination of small molecular substances, ITT
Average potassium reduction (%), V0 to V2

## Slide 17
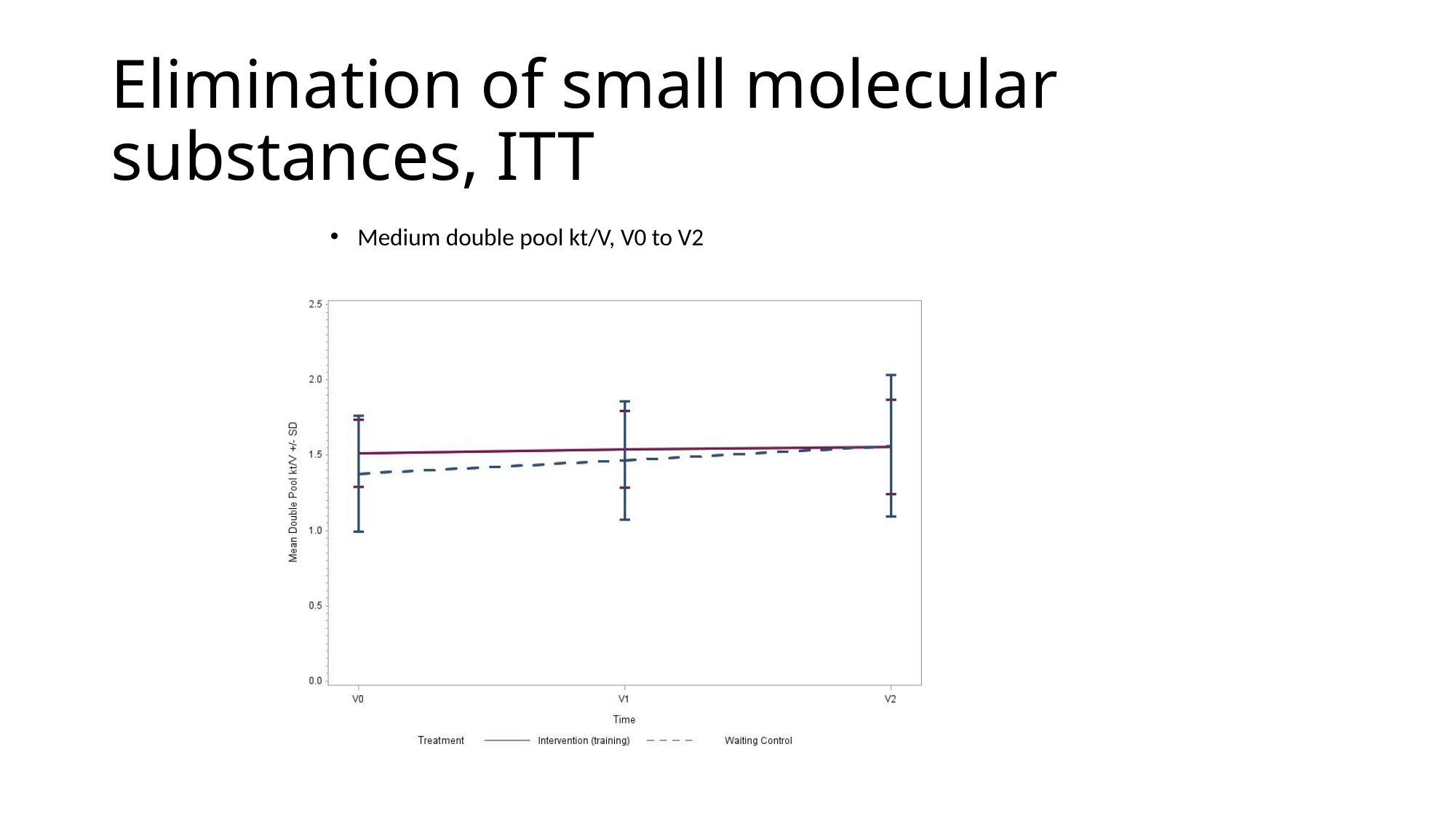

# Elimination of small molecular substances, ITT
Medium double pool kt/V, V0 to V2

## Slide 18
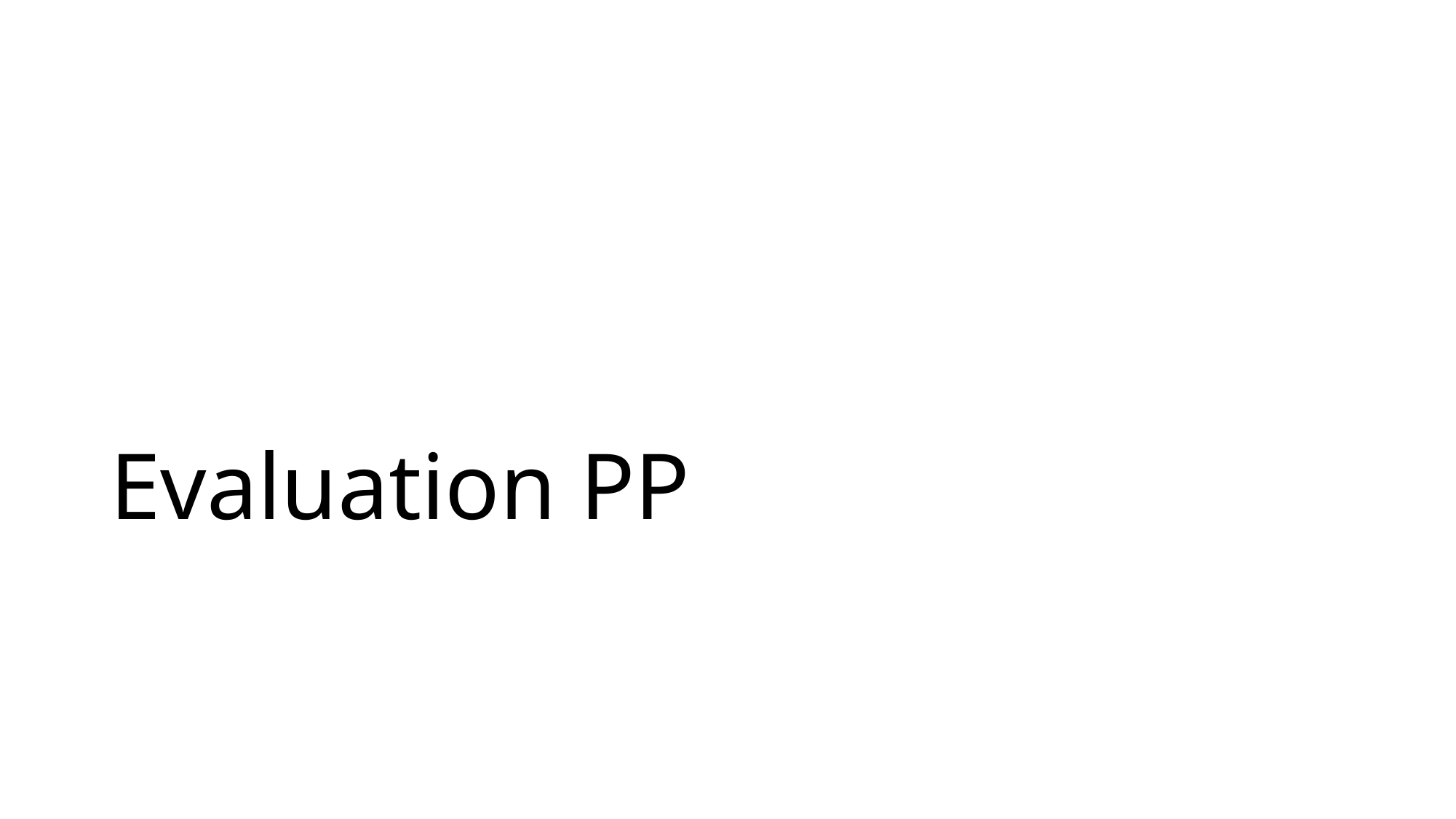

# Evaluation PP

## Slide 19
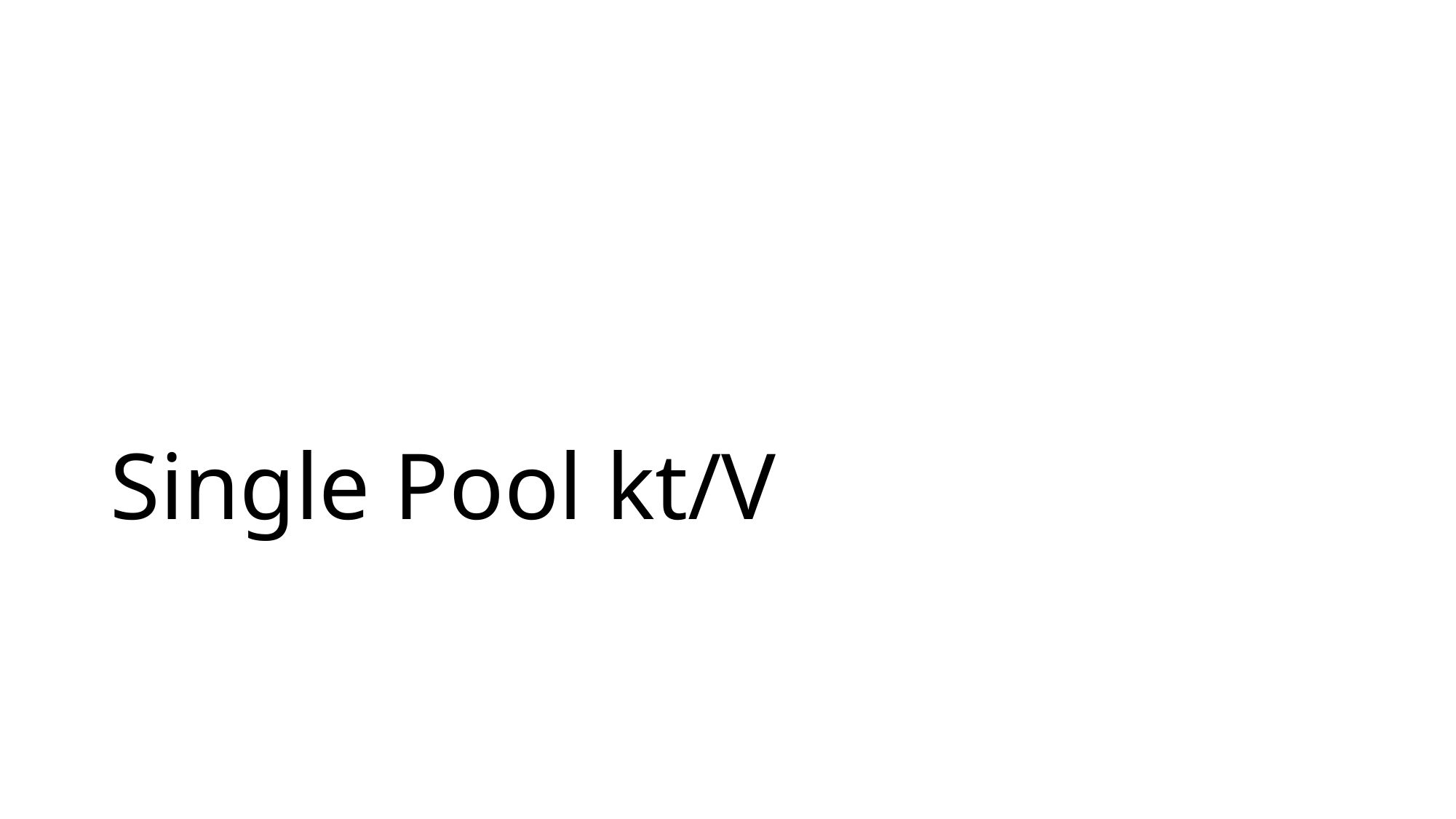

# Single Pool kt/V

## Slide 20
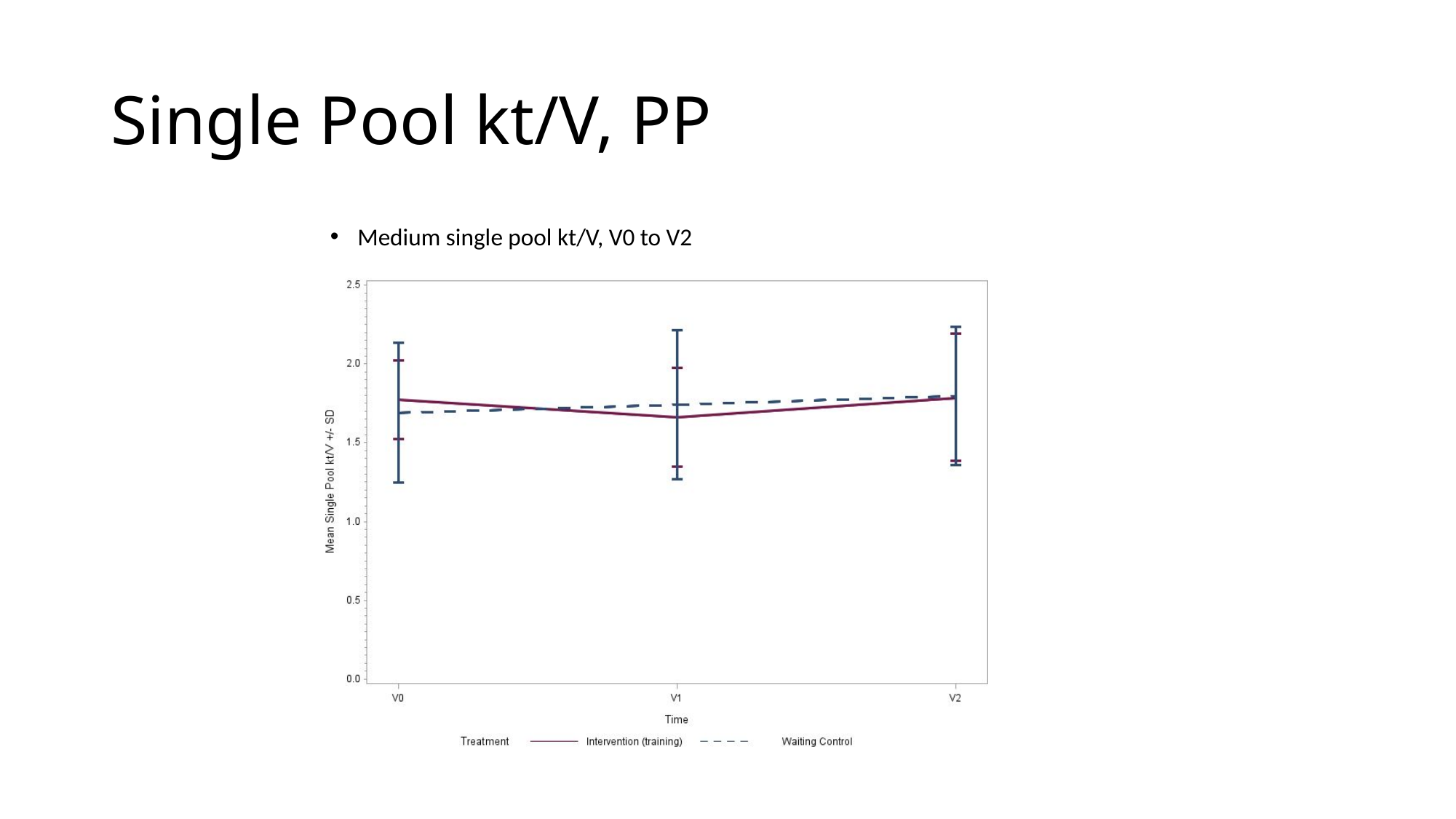

# Single Pool kt/V, PP
Medium single pool kt/V, V0 to V2

## Slide 21
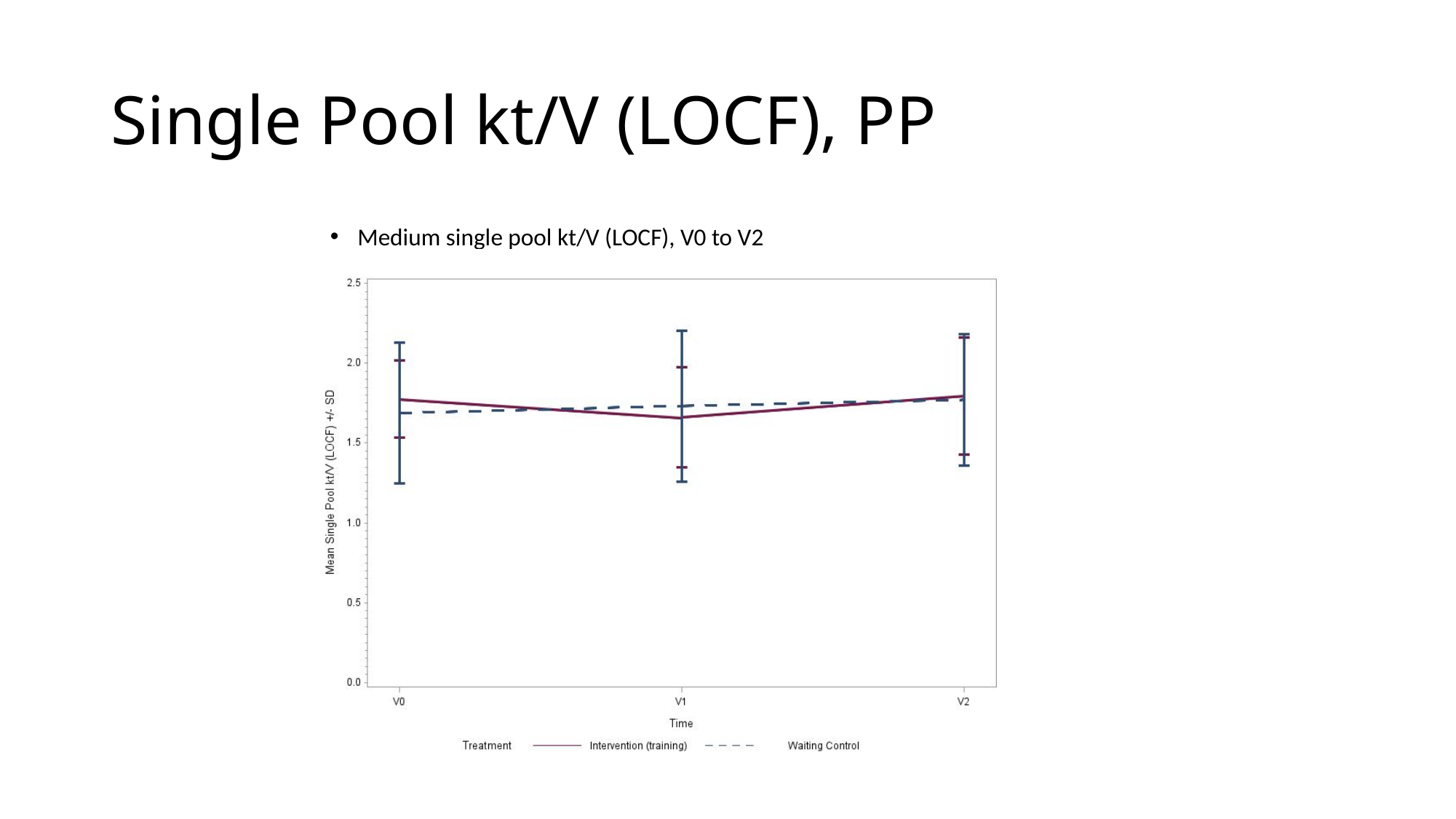

# Single Pool kt/V (LOCF), PP
Medium single pool kt/V (LOCF), V0 to V2

## Slide 22
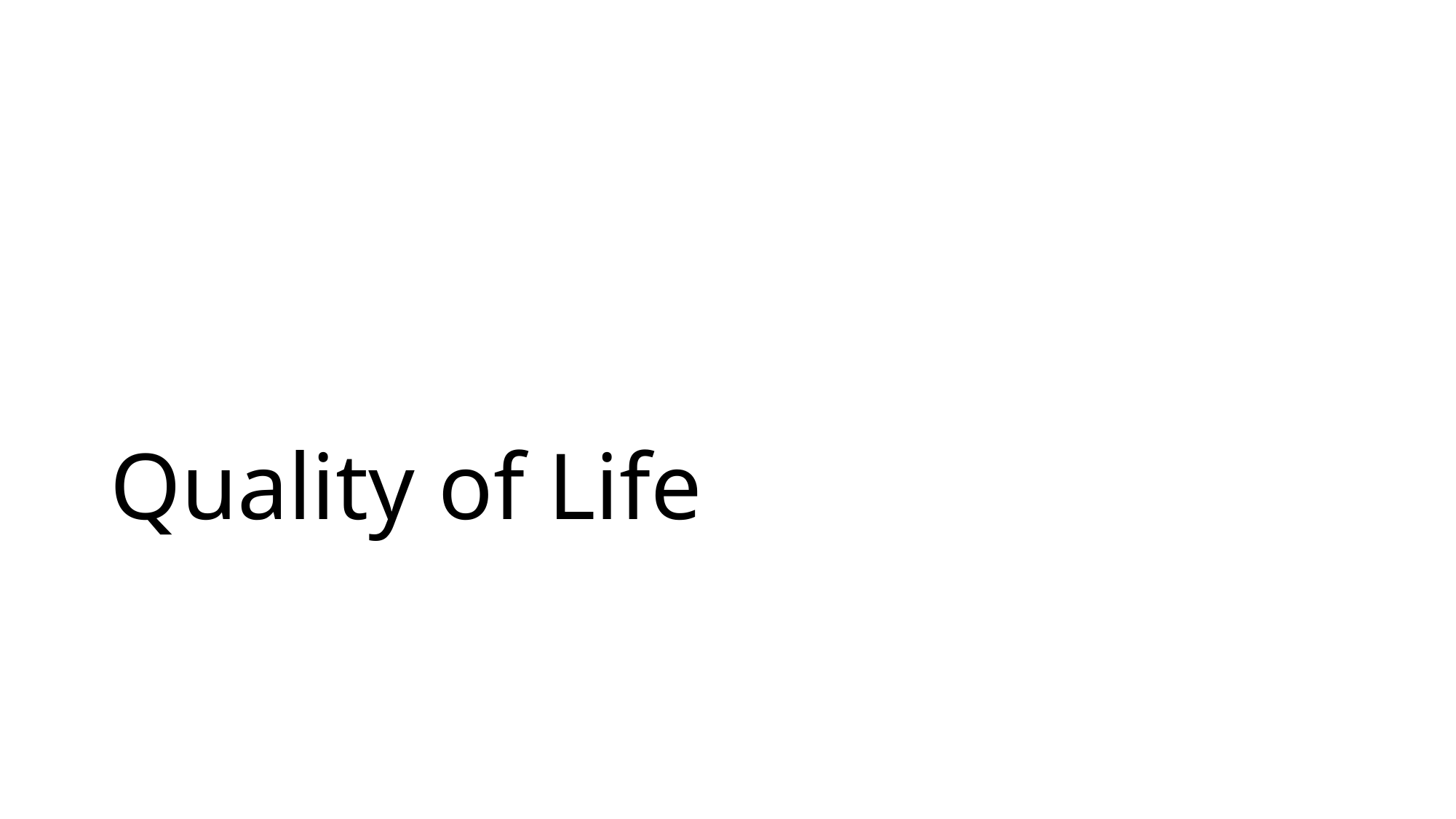

# Quality of Life

## Slide 23
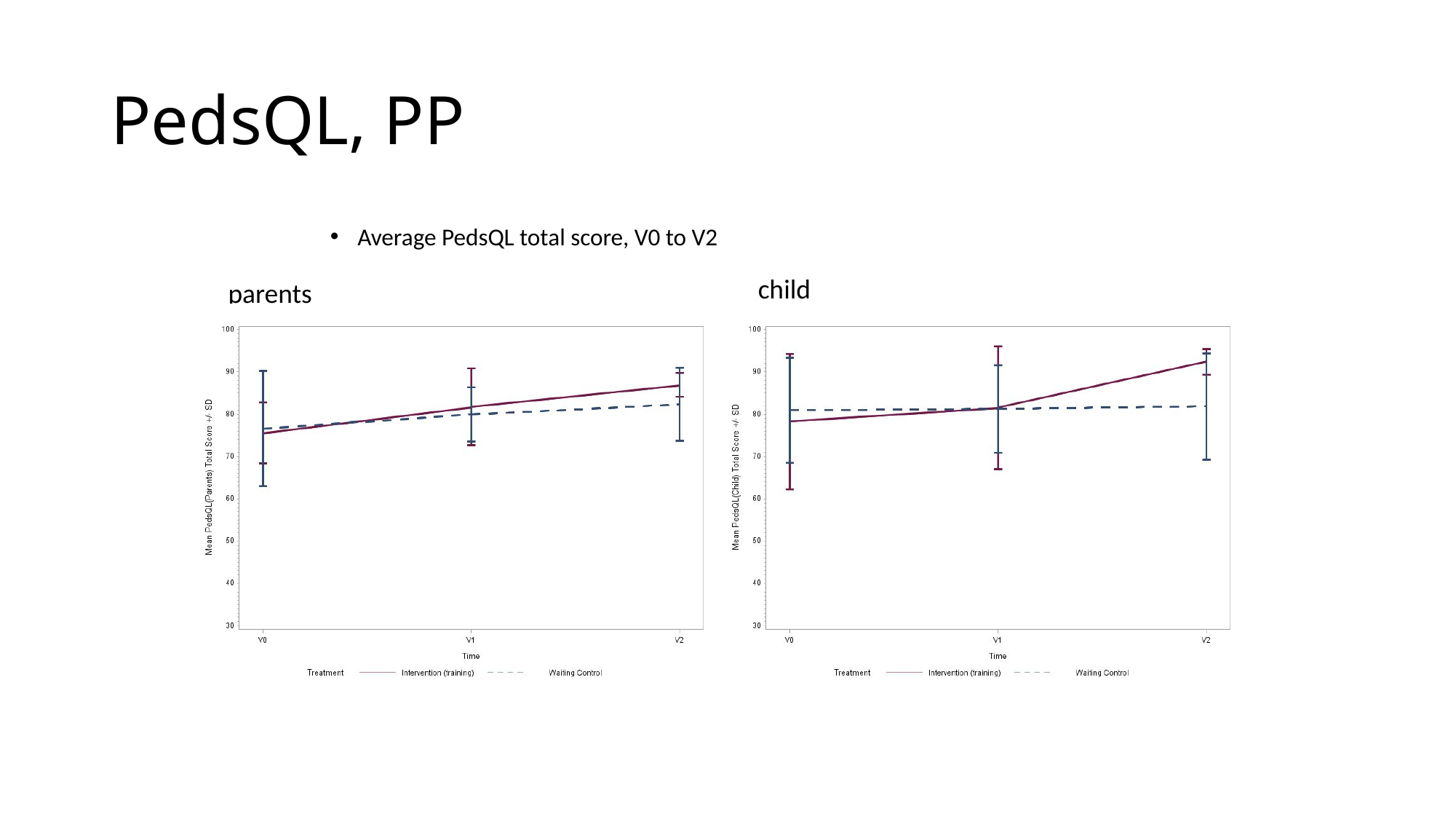

# PedsQL, PP
Average PedsQL total score, V0 to V2
child
parents

## Slide 24
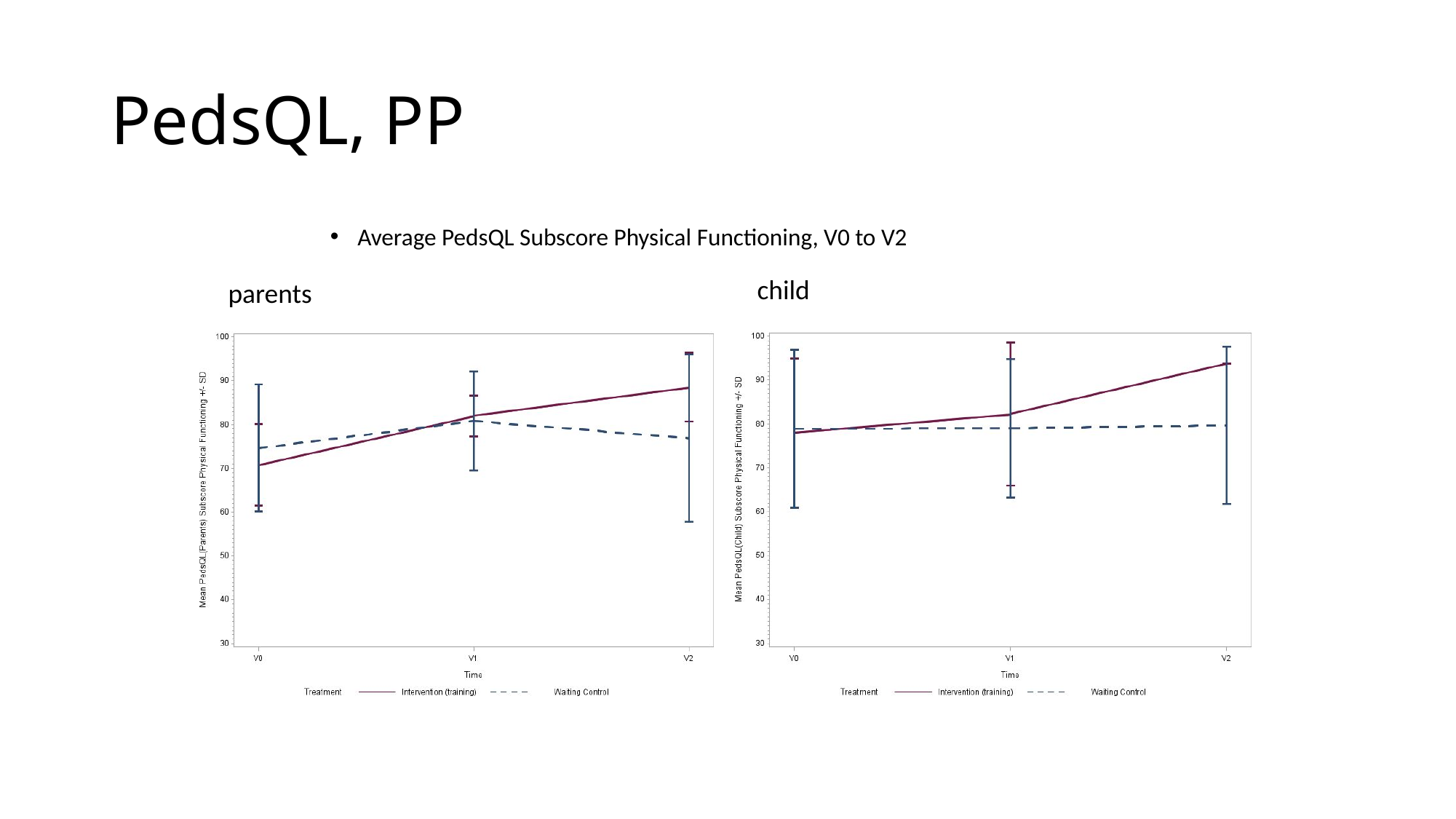

# PedsQL, PP
Average PedsQL Subscore Physical Functioning, V0 to V2
child
parents

## Slide 25
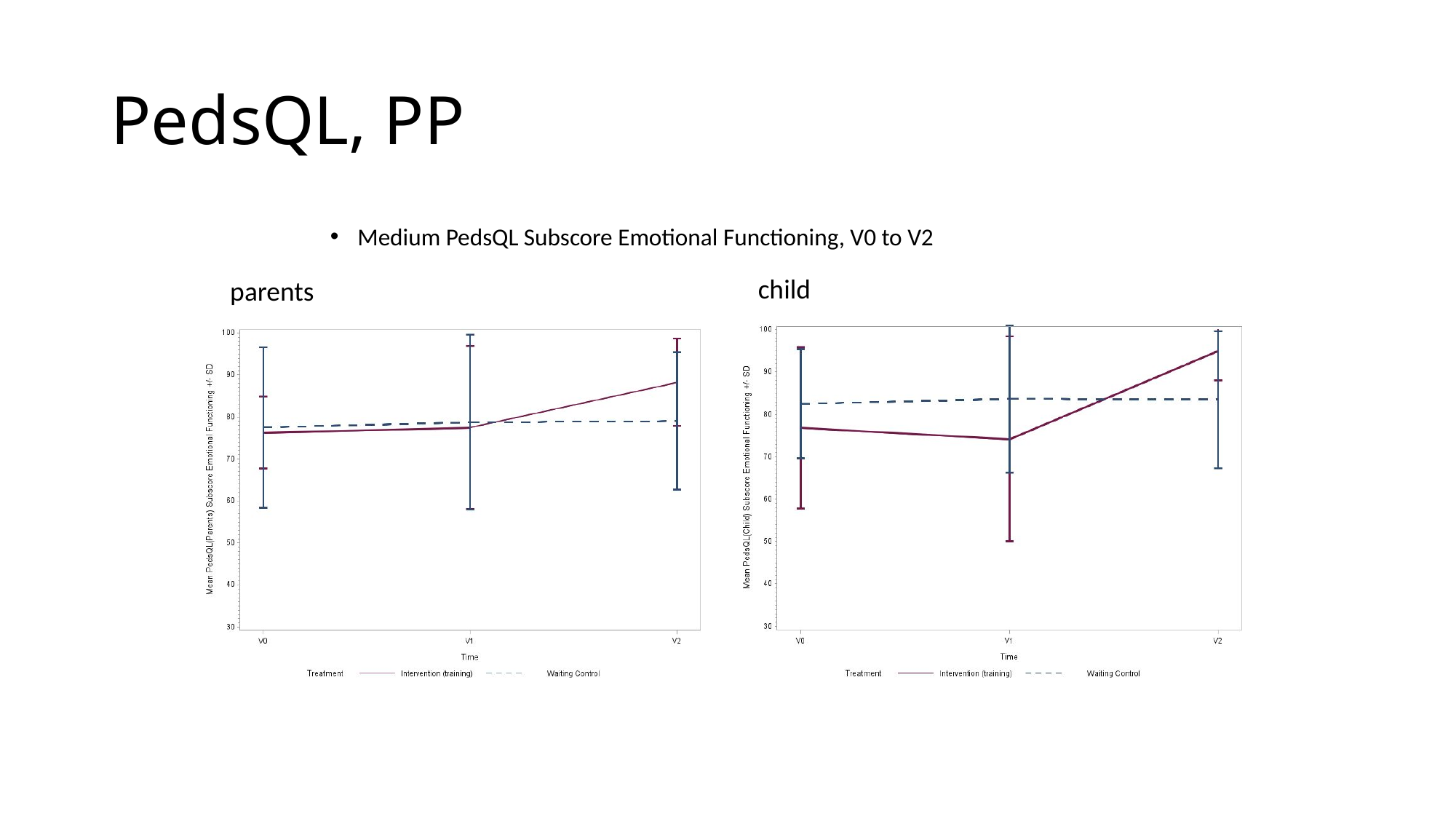

# PedsQL, PP
Medium PedsQL Subscore Emotional Functioning, V0 to V2
child
parents

## Slide 26
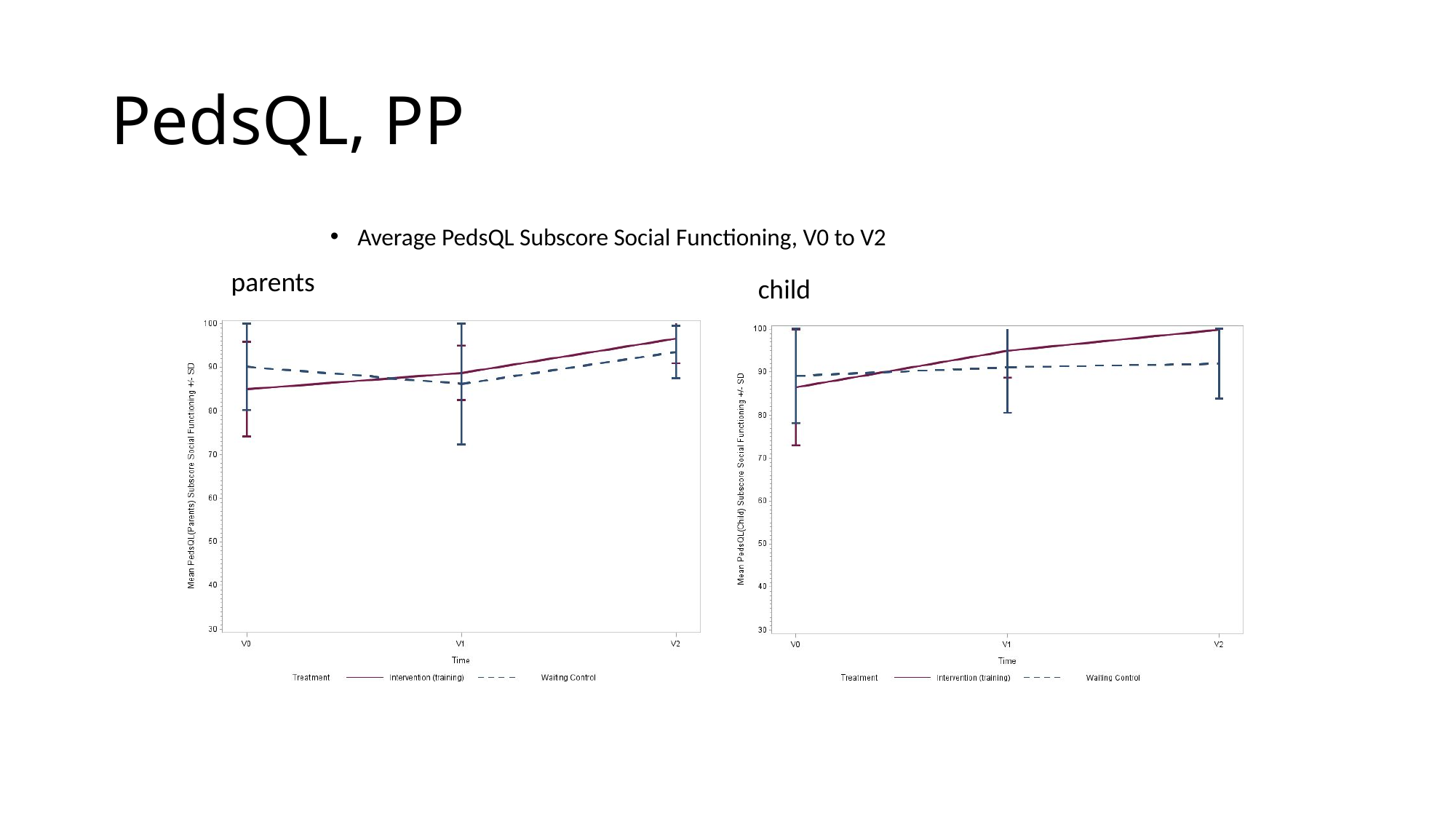

# PedsQL, PP
Average PedsQL Subscore Social Functioning, V0 to V2
parents
child

## Slide 27
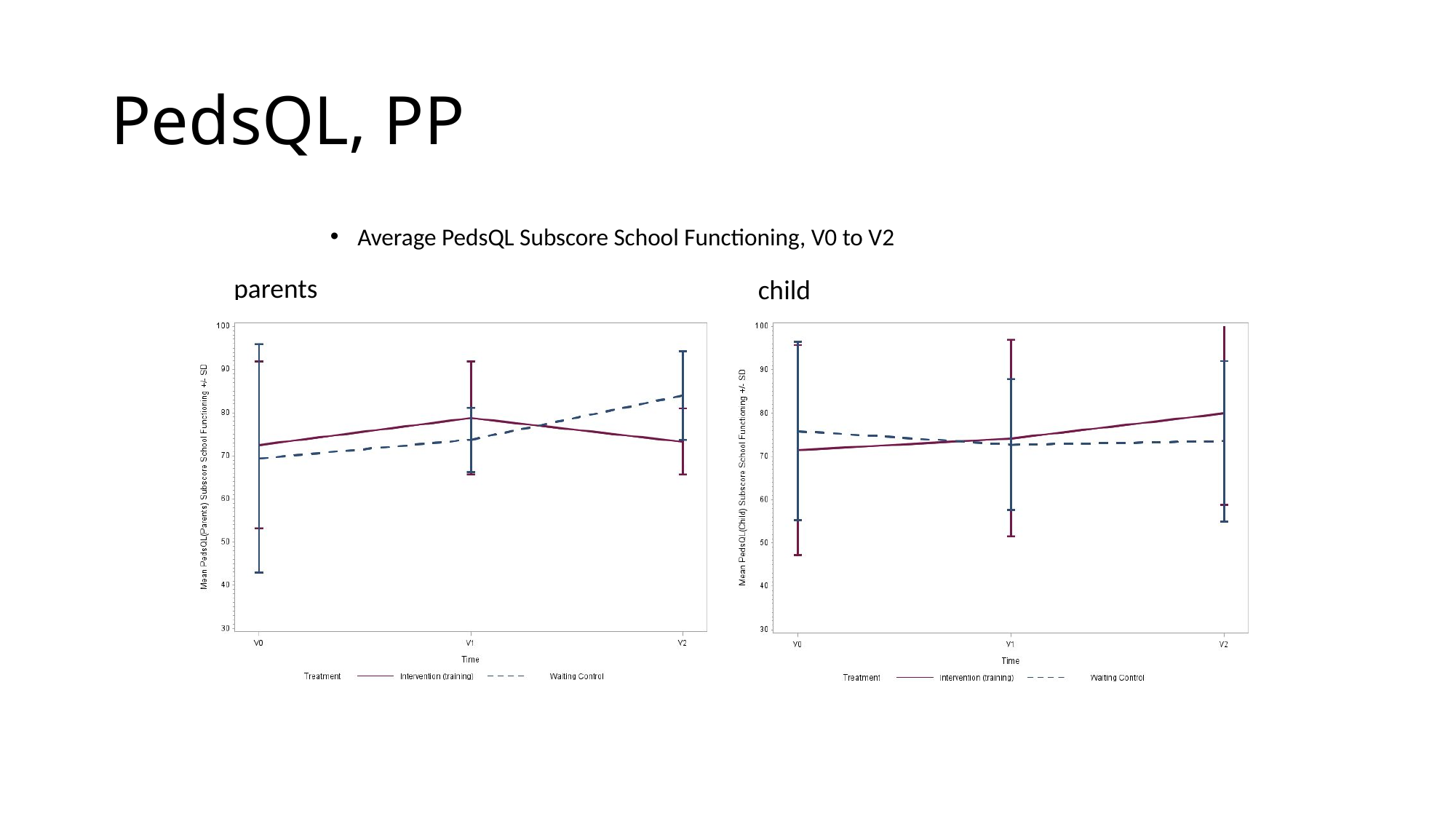

# PedsQL, PP
Average PedsQL Subscore School Functioning, V0 to V2
parents
child

## Slide 28
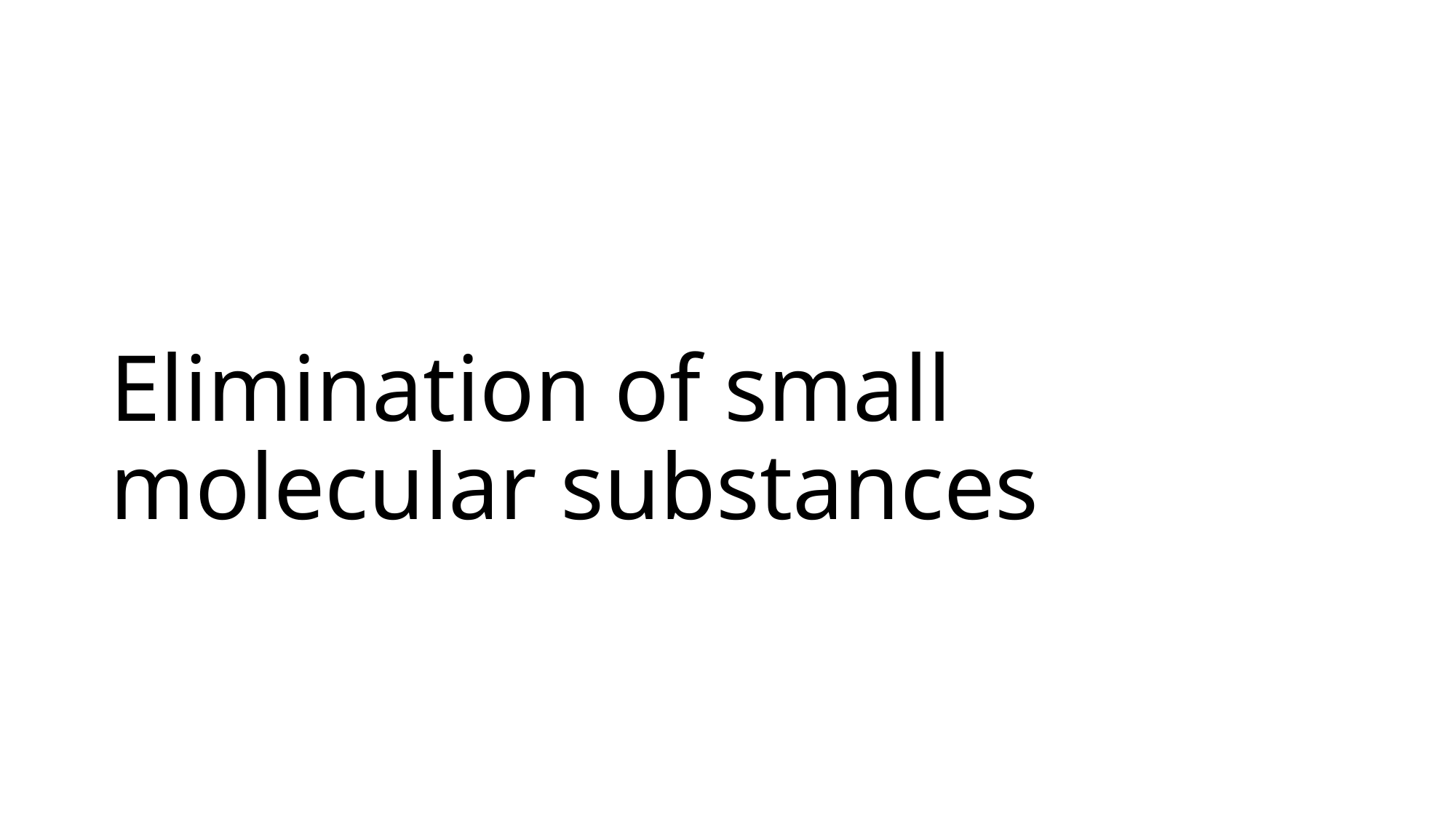

# Elimination of small molecular substances

## Slide 29
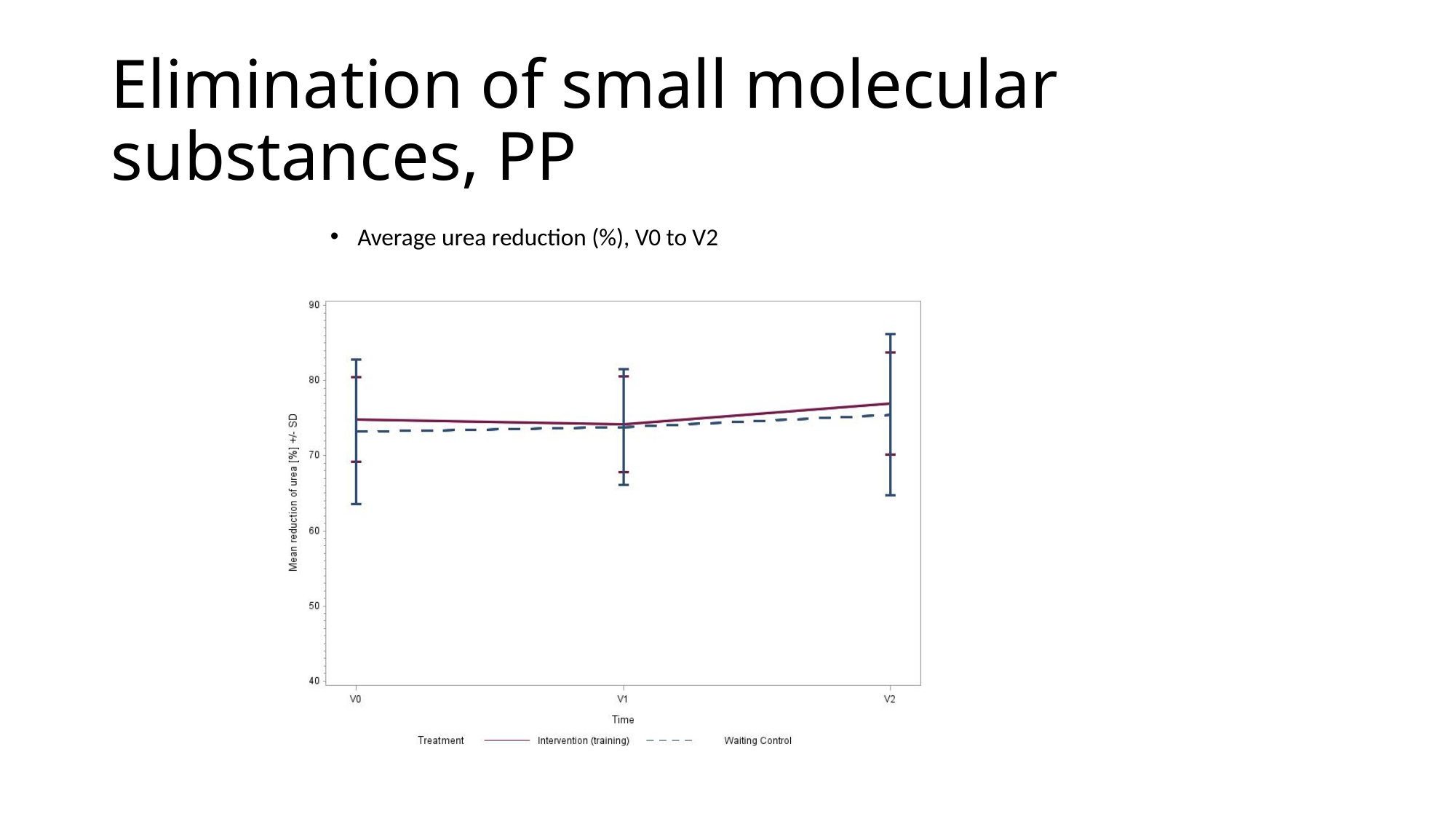

# Elimination of small molecular substances, PP
Average urea reduction (%), V0 to V2

## Slide 30
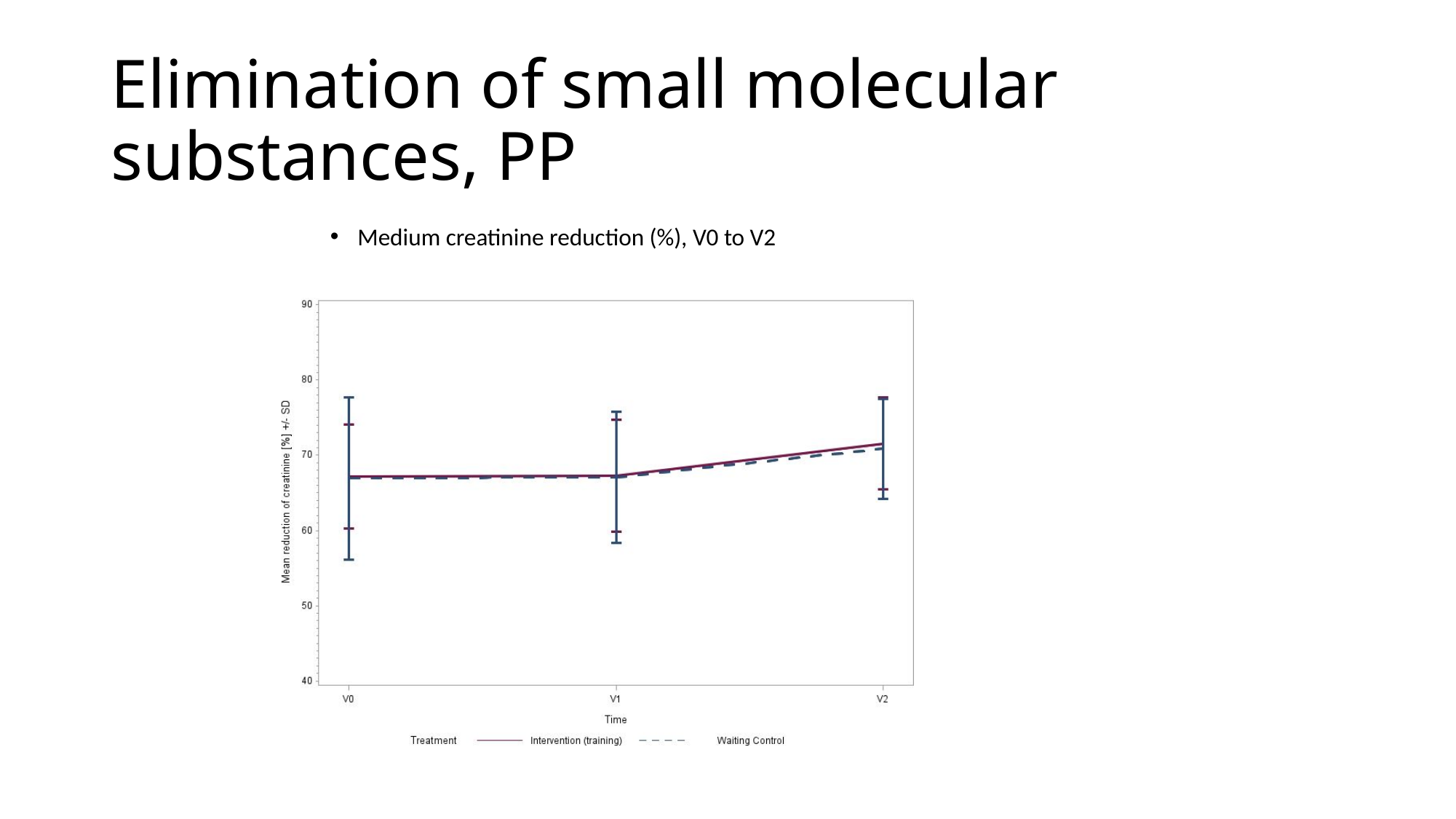

# Elimination of small molecular substances, PP
Medium creatinine reduction (%), V0 to V2

## Slide 31
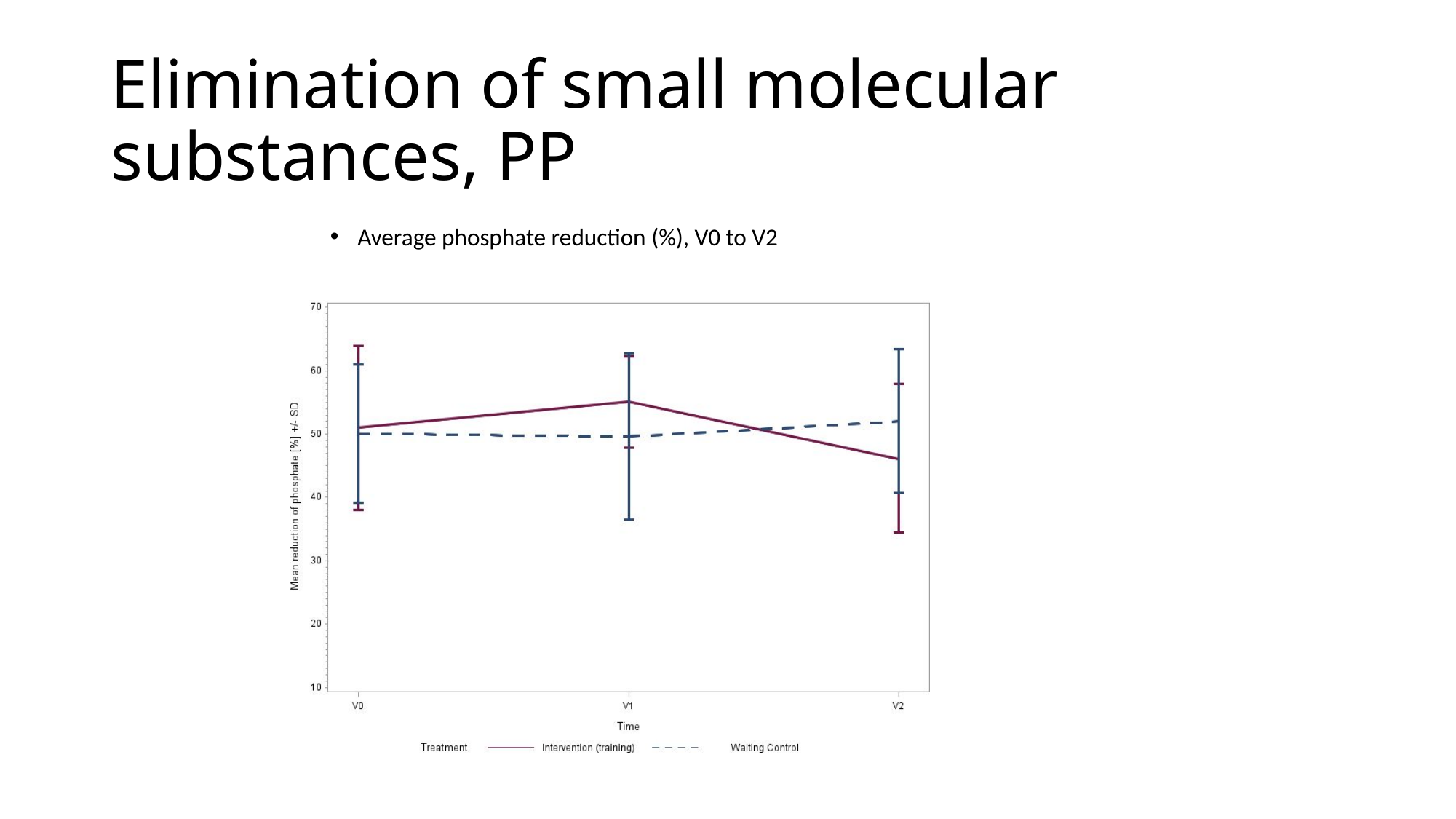

# Elimination of small molecular substances, PP
Average phosphate reduction (%), V0 to V2

## Slide 32
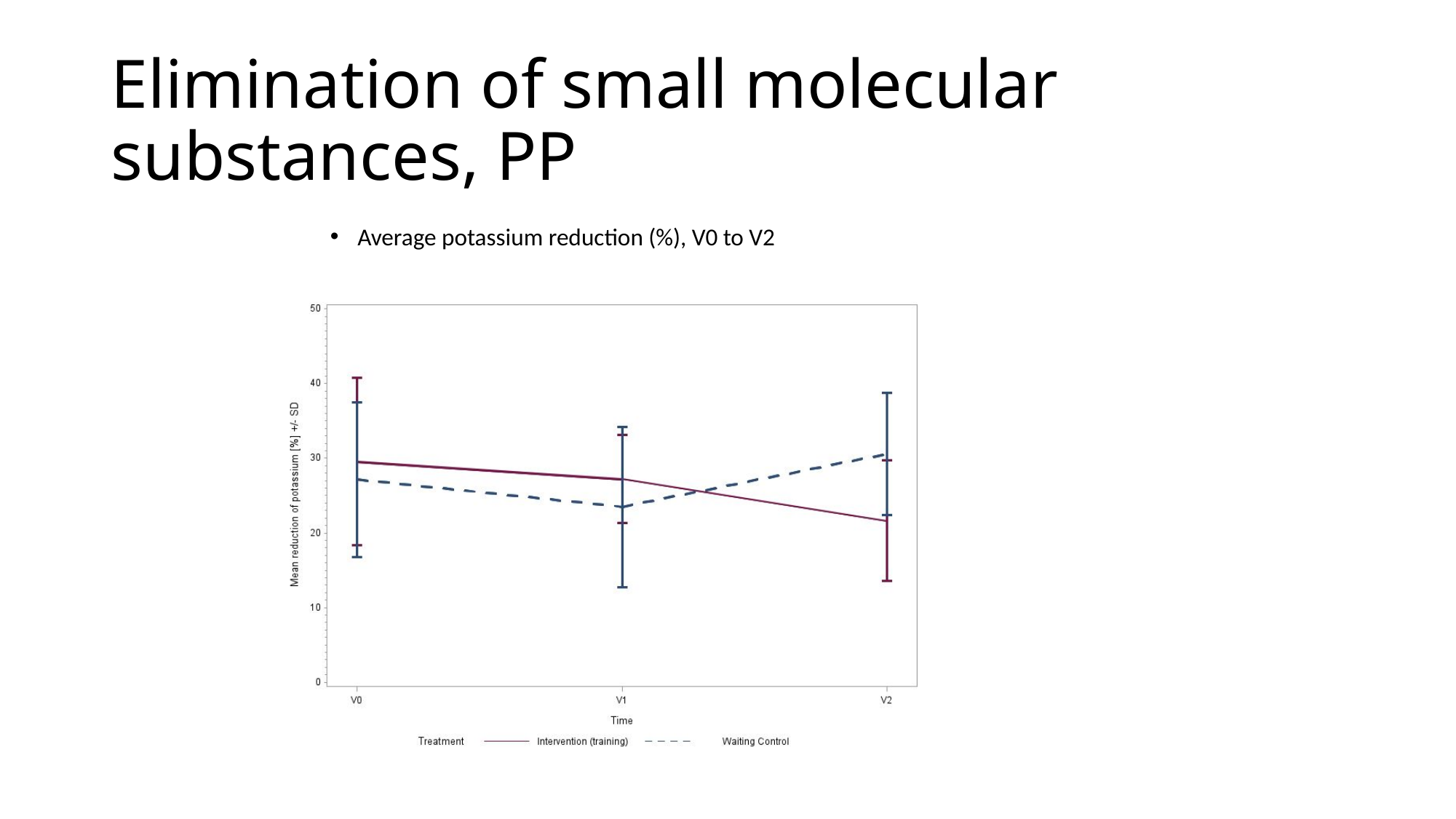

# Elimination of small molecular substances, PP
Average potassium reduction (%), V0 to V2

## Slide 33
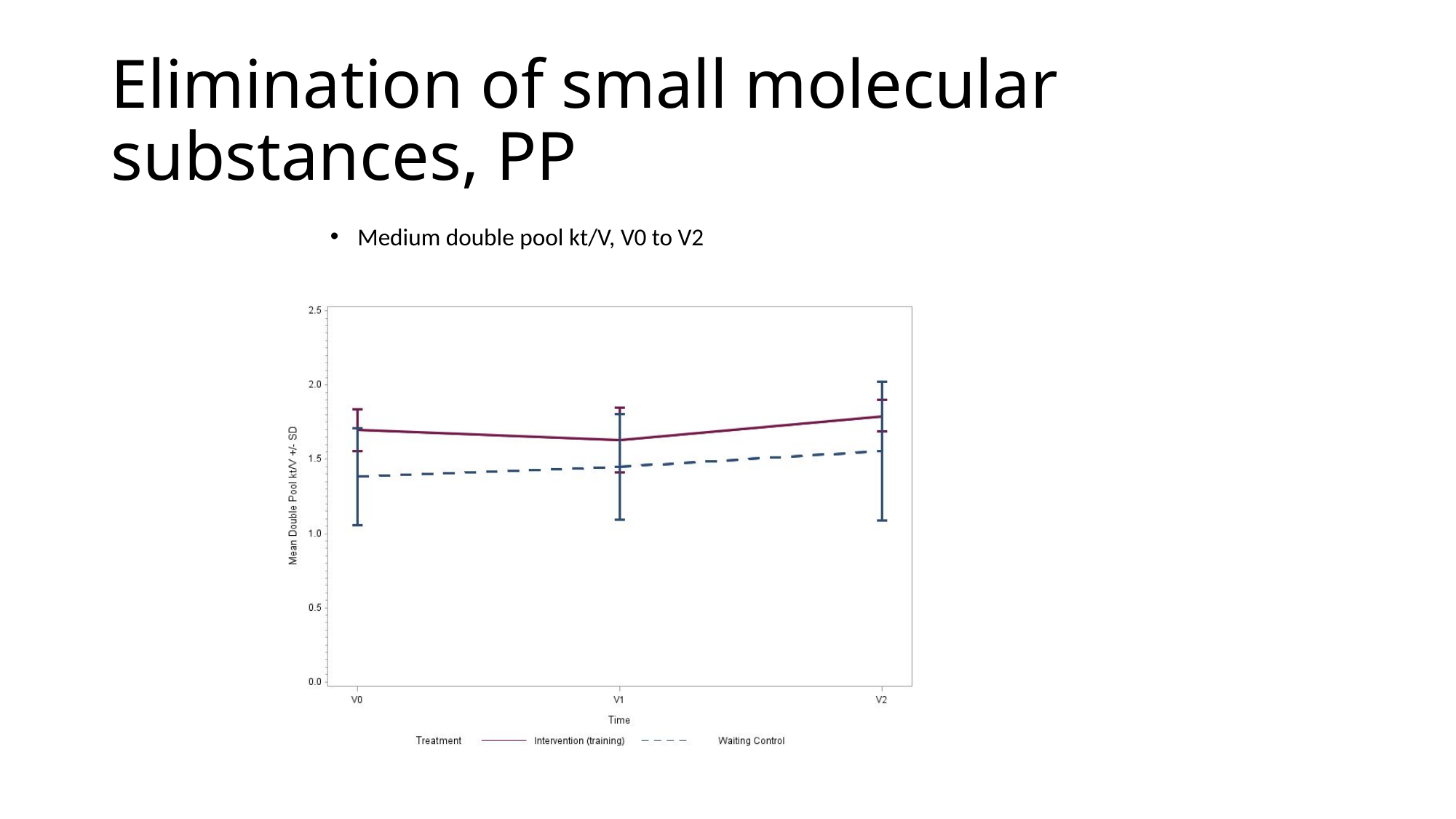

# Elimination of small molecular substances, PP
Medium double pool kt/V, V0 to V2

## Slide 34
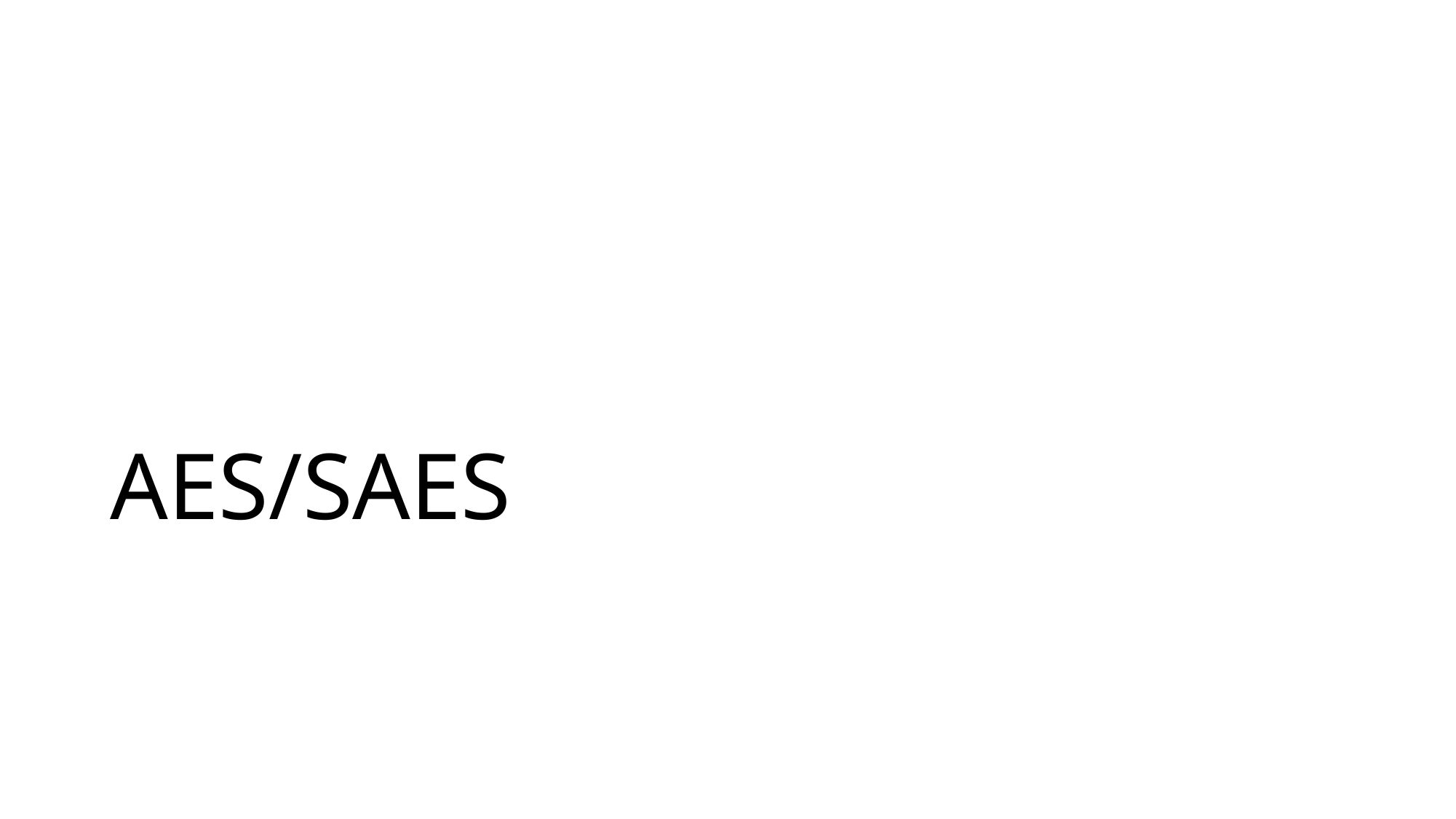

# AES/SAES

## Slide 35
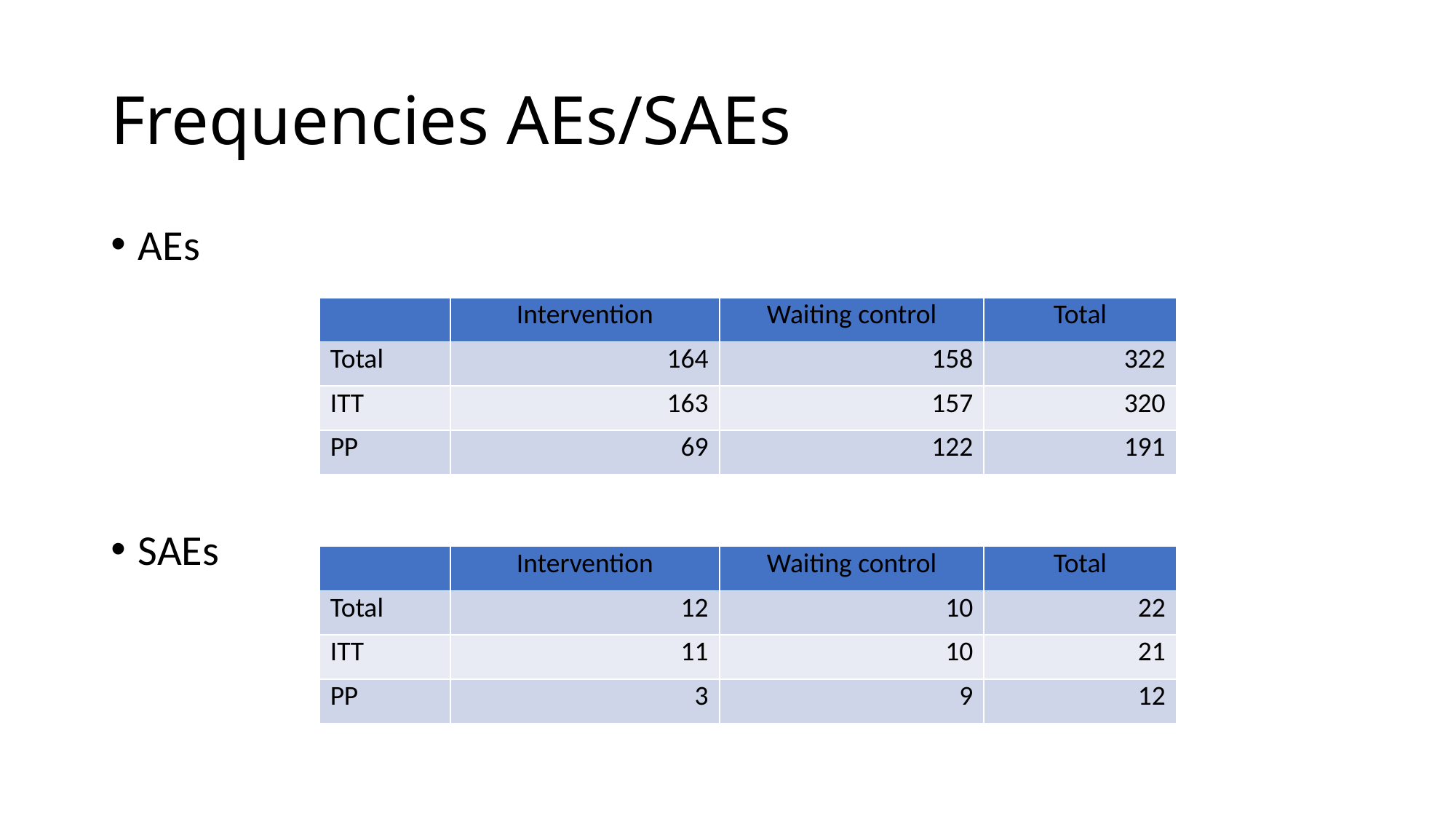

# Frequencies AEs/SAEs
AEs
SAEs
| | Intervention | Waiting control | Total |
| --- | --- | --- | --- |
| Total | 164 | 158 | 322 |
| ITT | 163 | 157 | 320 |
| PP | 69 | 122 | 191 |
| | Intervention | Waiting control | Total |
| --- | --- | --- | --- |
| Total | 12 | 10 | 22 |
| ITT | 11 | 10 | 21 |
| PP | 3 | 9 | 12 |

## Slide 36
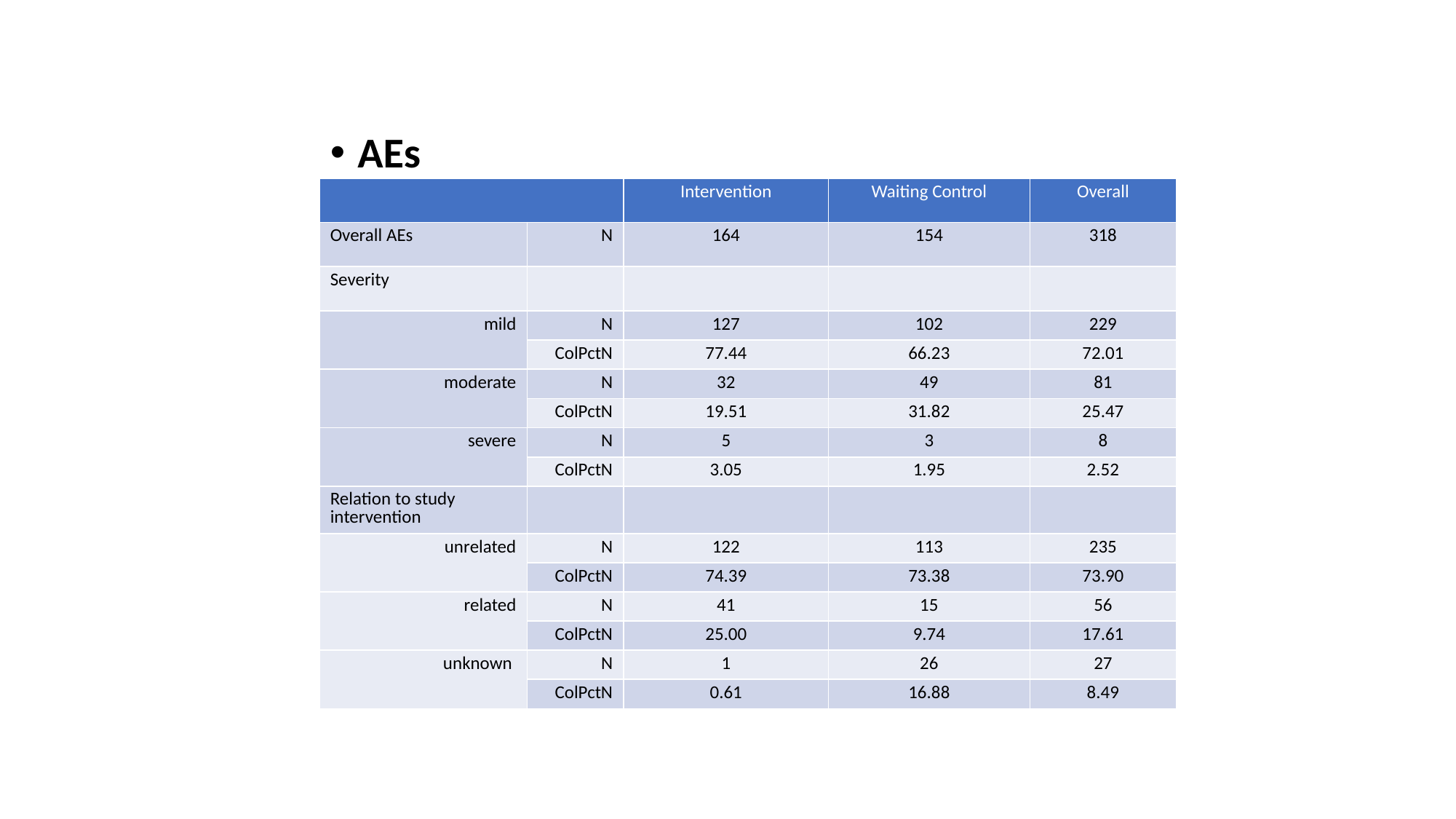

AEs
| | | Intervention | Waiting Control | Overall |
| --- | --- | --- | --- | --- |
| Overall AEs | N | 164 | 154 | 318 |
| Severity | | | | |
| mild | N | 127 | 102 | 229 |
| | ColPctN | 77.44 | 66.23 | 72.01 |
| moderate | N | 32 | 49 | 81 |
| | ColPctN | 19.51 | 31.82 | 25.47 |
| severe | N | 5 | 3 | 8 |
| | ColPctN | 3.05 | 1.95 | 2.52 |
| Relation to study intervention | | | | |
| unrelated | N | 122 | 113 | 235 |
| | ColPctN | 74.39 | 73.38 | 73.90 |
| related | N | 41 | 15 | 56 |
| | ColPctN | 25.00 | 9.74 | 17.61 |
| unknown | N | 1 | 26 | 27 |
| | ColPctN | 0.61 | 16.88 | 8.49 |

## Slide 37
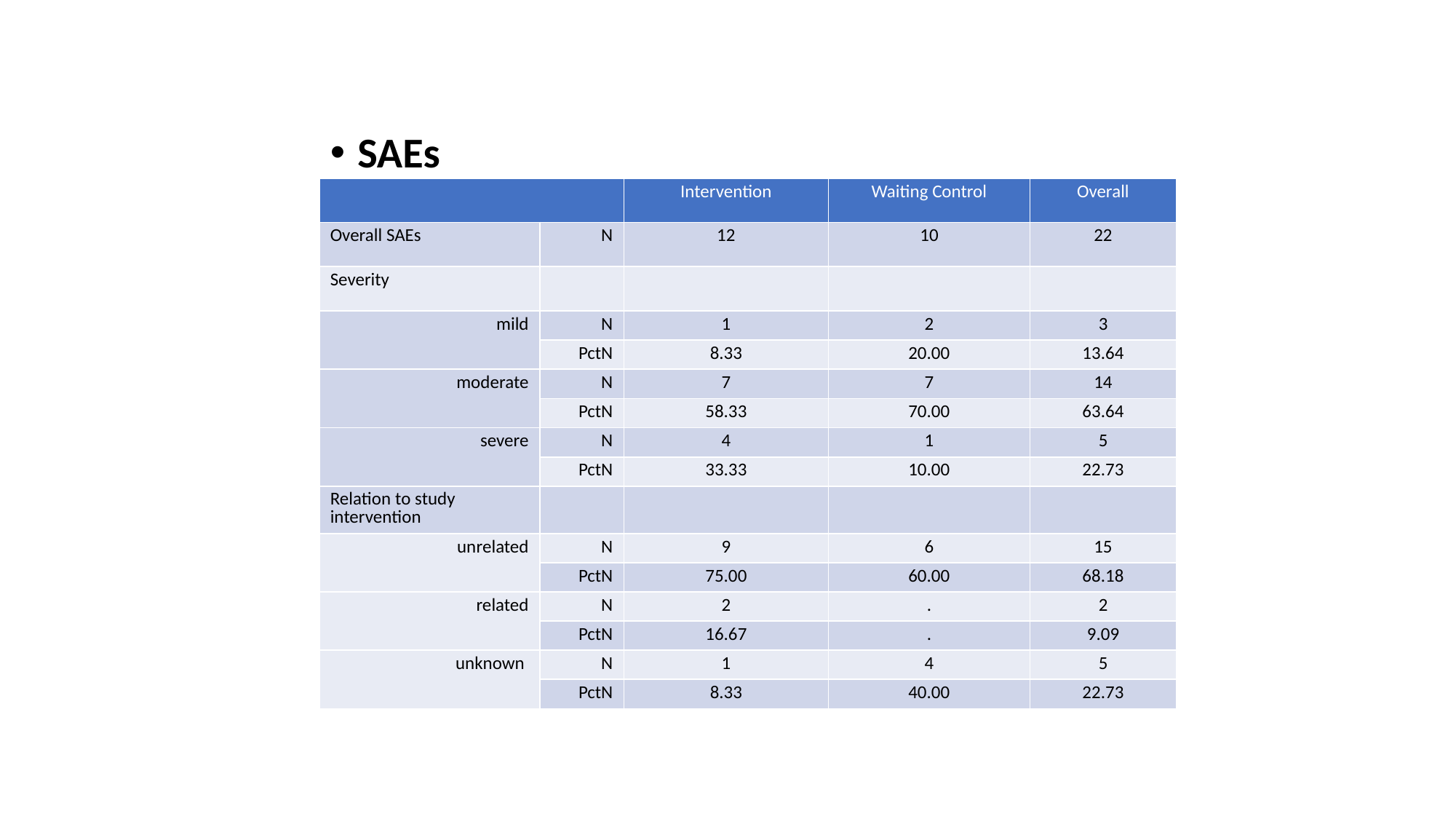

SAEs
| | | Intervention | Waiting Control | Overall |
| --- | --- | --- | --- | --- |
| Overall SAEs | N | 12 | 10 | 22 |
| Severity | | | | |
| mild | N | 1 | 2 | 3 |
| | PctN | 8.33 | 20.00 | 13.64 |
| moderate | N | 7 | 7 | 14 |
| | PctN | 58.33 | 70.00 | 63.64 |
| severe | N | 4 | 1 | 5 |
| | PctN | 33.33 | 10.00 | 22.73 |
| Relation to study intervention | | | | |
| unrelated | N | 9 | 6 | 15 |
| | PctN | 75.00 | 60.00 | 68.18 |
| related | N | 2 | . | 2 |
| | PctN | 16.67 | . | 9.09 |
| unknown | N | 1 | 4 | 5 |
| | PctN | 8.33 | 40.00 | 22.73 |
